# Viscous peeling of a nanosheet†

**DOI:** 10.1039/d1sm01743h

**Published:** 2022-05-05

**Authors:** Adyant Agrawal, Simon Gravelle, Catherine Kamal, Lorenzo Botto

**Affiliations:** School of Engineering and Material Science, Queen Mary University of London London UK; Process and Energy Department, 3ME Faculty of Mechanical, Maritime and Materials Engineering, TU Delft Delft The Netherlands l.botto@tudelft.nl

## Abstract

Combining molecular dynamics (MD) and continuum simulations, we study the dynamics of propagation of a peeling front in a system composed of multilayered graphene nanosheets completely immersed in water. Peeling is induced by lifting one of the nanosheet edges with an assigned pulling velocity normal to the flat substrate. Using MD, we compute the pulling force as a function of the pulling velocity, and quantify the viscous resistance to the advancement of the peeling front. We compare the MD results to a 1D continuum model of a sheet loaded with modelled hydrodynamic loads. Our results show that the viscous dependence of the force on the velocity is negligible below a threshold velocity. Above this threshold, the hydrodynamics is mainly controlled by the viscous resistance associated to the flow near the crack opening, while lubrication forces are negligible owing to the large hydrodynamic slip at the liquid-solid boundary. Two dissipative mechanisms are identified: a drag resistance to the upward motion of the edge, and a resistance to the gap opening associated to the curvature of the flow streamlines near the entrance. Surprisingly, the shape of the sheet was found to be approximately independent of the pulling velocity even for the largest velocities considered.

## Abstract 2

We carry out molecular dynamics of peeling of a graphene nanosheet from a flat substrate, for the case in which the system is completely immersed in water. Entrance effects are shown to dominate the dynamics of the peeling front.

## Graphical abstract

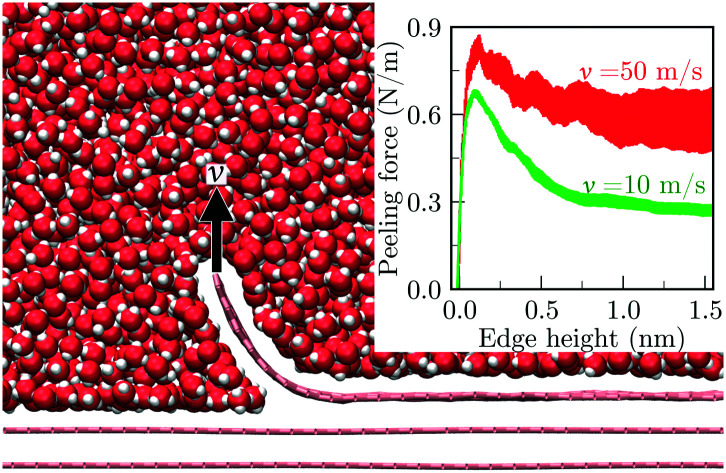

## 1 Introduction

Two-dimensional (2D) nanomaterials, such as graphene, boron nitride, and molybdenum disulfide, are flexible structures of atomic thickness that can bend like a sheet of paper when exposed to sufficiently large forces. When a thin sheet is bound to a substrate by adhesion, external forces can induce peeling of the sheet, a complex phenomenon involving a competition between adhesive, bending, and dissipative forces.^1–8^ The peeling of thin structures from rigid or soft substrates has received increasing attention from the soft matter community for its connections with soft wetting^9^ and its many applications in biology and engineering.^10–12^ However, the specific properties of 2D materials,^13^ such as low bending rigidity and unusual surface properties, rise questions on the validity of current models, designed for macroscopic sheets, when applied to the description of the peeling of 2D materials.

In many applications, peeling of 2D materials occurs in the presence of a liquid.^3,14–17^ For instance, in liquid-phase exfoliation processes for the large-scale production of graphene, colloidal microparticles of graphite are suspended in suitable liquid solvents (*e.g.* water, NMP), and shear is applied to the fluid–solid mixture until single- or few-layer graphene nanosheets detach from the ‘mother’ graphite microparticles.^14,15^ Fluids can also affect peeling of 2D materials in applications that do not involve bulk liquid solution, such as the transfer of 2D materials between substrates, where water is often present owing to condensation from the surrounding air.^18,19^ While the mechanics of peeling of 2D materials in vacuum or air has been studied extensively through theory and experiments,^2,20–23^ peeling of 2D materials in liquids is a new subject.^24–26^ Peeling off 2D materials from a substrate or a stack of other sheets in the presence of a liquid requires initially lifting ‘flaps’,^27^ which can have nanometric length at the initial stages of the peeling process. The removal by peeling of 2D materials in liquids brings about a new set of scientific challenges, particularly considering that most available theories have been developed to explain the results of macroscopic applications such as adhesive tests or hydraulic fracturing.^1,3–5,8^

The presence of a liquid primarily has two effects on the peeling of thin flexible sheets from a flat substrate: the liquid can alter directly the magnitude of the adhesion force between the bonded layers, by modifying *e.g.* the Hamaker constant;^24^ and the liquid flow induces viscous forces on the peeled layer, which in turn affect the value of the peeling force.^28^ For macroscopic sheets, the resistance to the motion of the peeling front originates mainly from lubrication forces.^4^ These forces emerge from the motion of the peeled layer in the direction normal to the substrate, which produces a parabolic flow in the small gap between the sheets. The lubrication forces associated to this flow are extremely sensitive to the boundary condition: even small deviations from the no-slip condition can reduce lubrication forces substantially, and this effect is most marked when the slip length is comparable to the gap size.^29–32^ This raises the question as to what mechanisms determine the resistance to motion of the peeling front when the slip length is comparable or larger than system size, *i.e.* the maximum gap height or the typical crack length.

**Fig. 1:**
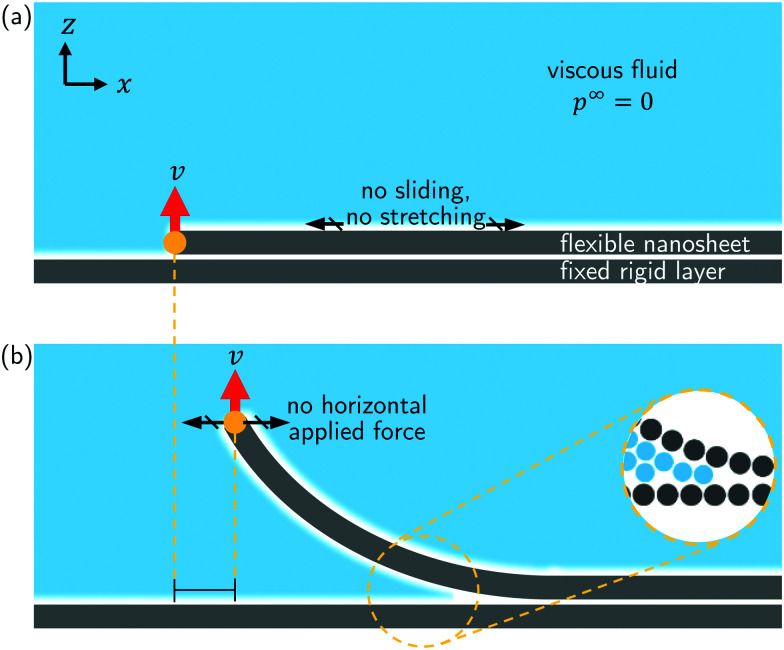
Illustration of the physical problem. (a) At time *t* = 0, a semi-infinite flexible nanosheet is completely bound to a rigid horizontal layer *via* van der Waals forces. The surrounding fluid does not enter the small gap between the sheets yet. The sheet is inextensible and cannot slide on the horizontal surface. (b) At time *t* ≥ 0, the edge of the flexible nanosheet is pulled upwards with a velocity *v*. The horizontal component of the applied force is zero (free horizontal sliding). The magnified view of the peeling front shows that fluid molecules penetrate only until the point where the separation between the nanosheet and the horizontal layer is too small to accommodate a fluid molecule.

In this paper, we combine Molecular Dynamics (MD) and continuum modelling to study the peeling of short (few nanometers) graphene layers from a rigid substrate, for the case in which the maximum gap height and maximum crack length are smaller than the slip length characterising the hydrodynamic boundary condition at the solid-liquid interface.^33^Fig. 1 illustrates the physical configuration simulated. In our problem, the rigid substrate simulated with MD is composed of a stack of graphene layers, a configuration motivated by applications to liquid-phase exfoliation of graphite microparticles. The peeled layer and the substrate are in contact with a liquid solvent (Fig. 1). As solvent, for this study we choose water because of the quality of published data on graphene–water interaction^29,34,35^ and because water in MD displays Newtonian behaviour even at large shear rates^36,37^ (in the ESI,† we also show results for NMP as solvent). Peeling is induced by displacing the edge of the flexible graphene layer with an assigned velocity *v* normal to the substrate. The force on the edge is measured. The objectives of the paper are to quantify the features of the force-velocity curve, and get insights into dissipative mechanisms controlling the dynamics of the peeling front. The slip length *λ* characterising the surface of pristine graphene in water and many other liquid solvents is rather large.^38^ Slip lengths of 
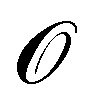
(10 nm) have been reported for water-graphene interface both from experiments^39,40^ and *ab initio* molecular dynamics methods.^41^ Therefore our MD simulation results, for which the crack length and maximum gap height are smaller than *λ*, cannot be explained by classical models based on lubrication theory. The paper therefore analyses alternative sources of viscous dissipation based on the comparison of MD results with different estimates for the viscous contribution to the pulling force.

Large slip lengths have been measured with many complex, structured fluids such as polymer melts, polymer solutions, colloidal suspensions, and colloidal gels.^42^ Slip is also significant in surfactant-covered solid surfaces when a high shear stress is applied to the fluid,^43^ surfaces covered by polymer layers,^44^ flow of rarefied gasses in microchannels,^45^ and flow in nano-confined liquid systems.^46^ The results of our investigation may thus have more general implications for soft matter research than our current focus on graphene may suggest. Our work is also relevant to understanding the effect of hydrodynamic forces in adhesion measurements. For example, in the measurement of the adhesion between surfaces in contact with high-viscosity ionic liquids, viscous forces are an important component of the force measured by a Surface Force Apparatus beyond a critical velocity that depends, among other parameters, on the slip length.^47^ The prediction of this velocity is an important practical question.

The structure of the paper is as follows. We first analyse MD simulation results to quantify the dependence of pulling force on velocity. We then develop a one-dimensional non-linear solid mechanics model based on the equation for the *elastica* to obtain insights into the relation between pulling force, pulling velocity and edge height. We then compare the results between MD and the model in the quasi-static limit, and later consider the velocity dependent case. For the velocity dependent case, we complement the results with finite-element COMSOL simulations for a simplified geometry. In these simulations, the solid–fluid momentum coupling is fully resolved, so we can extract quantities – such as the full profile of the pressure below the peeling sheet – that the one-dimensional *elastica* model alone cannot provide.

## 2 Results and discussion

### 2.1 Molecular dynamics

**Fig. 2:**
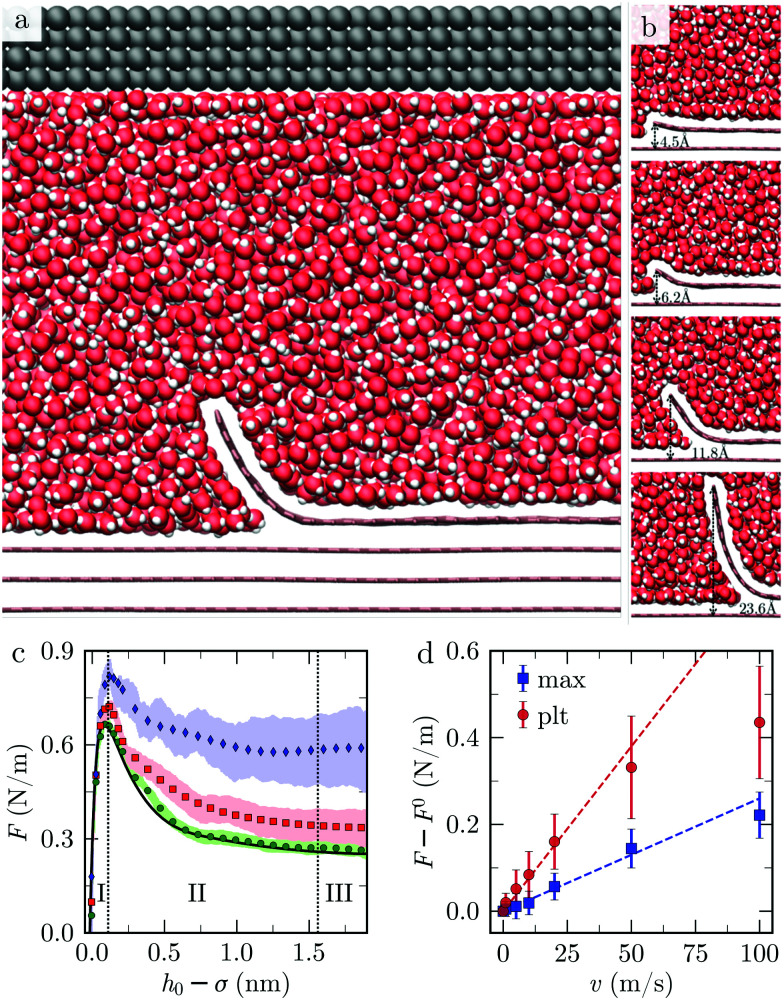
Analysis of Molecular Dynamics (MD) simulations. (a) Side view of the MD system with water in red and white, graphene in pink, and the rigid wall in gray. (b) Close-up view of the MD system for 4 values of the peeled height (*v* = 1 m s^−1^). (c) Peeling force as a function of edge height extracted from MD for different peeling velocities; *v* = 1 m s^−1^ (green disks), *v* = 10 m s^−1^ (red squares), and *v* = 50 m s^−1^ (blue lozenges). The colored areas correspond to the standard deviation, and the black full line to the static case (*v* = 0). *σ* = 3.4 Å is the equilibrium sheet height. (d) Dynamic peeling force minus the static peeling force (*F* − *F*(*v* = 0)) as a function of *v*. Red disks are *F*_plt_, *i.e.*, *F* calculated at *h*_0_ = 2 nm, and the blue squares are *F*_max_.

The peeling simulations of a single graphene nanosheet from a multilayer graphene surface in water are carried out using MD (LAMMPS software^48^). The TIP4P/2005 model^49^ is used for water and the AIREBO force field^50^ is used to model graphene, same as that used in literature to describe graphene-water systems.^26,38,46,51^ Additional details of the simulation are given in the ESI.† The initial configuration consists of a stack of 4 graphene layers. The three bottom layers are periodic along the e⃑_*x*_ and e⃑_*y*_ directions. The top layer is periodic along e⃑_*y*_, but shorter along the e⃑_*x*_ direction, thereby creating two edges. The graphene layers are in contact with water, and a piston wall^52^ is used to enclose the fluid in the e⃑_*z*_ direction and impose a pressure *p*_0_ = 1 atm (Fig. 2a). The length of simulation box is equal to 7.2 nm in e⃑_*x*_, 2.5 nm in e⃑_*y*_ and 8 nm in e⃑_*z*_, and the distance between the piston and the top graphene layer is approximately 4 nm.

The bottom three layers are maintained fixed, and the top layer is free to move. A velocity of magnitude *v* is applied along e⃑_*z*_ to one of the edges of the top layer. Velocities in the range of 1 to 100 m s^−1^ are used. Lower values for *v* are difficult to reach due to computational limitation. For values of *v* ≥ 100 m s^−1^, cavitation is observed (discussed further in Section 2.4). The other edge is maintained near its original position along e⃑_*x*_ by a harmonic potential. We make sure that restraining the motion of the top layer along e⃑_*x*_ has no impact on the final results by performing the same simulation in absence of the harmonic potential. We evaluate the sheet height along e⃑_*y*_, *h*(*x*), as the vertical distance between the centers of the carbon atoms of the deformable sheet and the sheet immediately under it. The equilibrium sheet height, *σ*, is measured to be ≈3.4 Å when the sheets are completely adhered. Starting from the equilibrium height (*σ*), we measure the distance *h*_0_ between the peeled edge and the fixed layer, and the force *F* (per unit width) along e⃑_*z*_ resisting peeling. We term *F* the peeling force and *h*_0_ the edge height. In addition to dynamic simulations, we also perform static simulations by imposing a constant value for *h*_0_ for 80 ps followed by an acquisition period of 120 ps.

The peeling force *F* was extracted from MD as a function of *h*_0_. We find that as *h*_0_ increases initially, *F* reaches a maximum, *F*_max_ (Fig. 2c). As *h*_0_ increases further, *F* decreases to a plateau value *F*_plt_. A similar trend was recently reported by Ouyang *et al.*^53^ in MD simulations of peeling a graphene nanoribbon off a hexagonal boron nitride monolayer. Qualitatively, the trend of *F vs. h*_0_ in Fig. 2c can be divided into three regions. In region I, *F* increases to a maximum *F*_max_; in region II, *F* slowly decreases as *h*_0_ increases; and in region III, *F* reaches a plateau value *F*_plt_. We observe this qualitative trend for 3 values of the peeling velocity (*v* = 1,10 and 50 m s^−1^.).

For *v* = 1 m s^−1^, *F* is approximately equal to the force *F*^0^ obtained from the steady-state simulation (*v* = 0). For *v* = 10 m s^−1^ and 50 m s^−1^, the dependence on peeling velocity is evident (Fig. 2c). To ascertain that the increase of the force with the velocity is originating from the viscous dissipation within the fluid, we performed a simulation in absence of fluid with *v* = 100 m s^−1^. This simulation showed no evident increase of the force with respect to the static case in vacuum (ESI,† Fig. S2). Both the characteristic maximum (*F*_max_) and plateau (*F*_plt_) values of the force increase with *v*. For *v* < 50 m s^−1^, the difference *F*–*F*^0^ between the total peeling force and the peeling force at steady state increases approximately linearly with *v* (Fig. 2d). We observe that *F*_plt_ depends more strongly on *v* than *F*_max_. For larger velocities, the rate of increase of *F* decreases. From Fig. 2d, we can estimate the order of magnitude of an effective friction coefficient *ξ*_eff_ ∼ (*F* − *F*_*v*=0_)/*v* using *F*_max_ and *F*_plt_ as two extremes. We find 2.6 mPa s ≤ *ξ*_eff_ ≤ 7.6 mPa s. We notice that *η*_eff_ has the same order of magnitude of the viscosity of water (*η* = 0.855 mPa s for the TIP4P/2005 water model used in this study^54^).

We compare snapshots of the system in the 3 regions of Fig. 2c. In region I the fluid does not intercalate between the adhered layers and no fluid molecules are present inside the crack (Fig. 2b). The slope of the sheet is small in this regime. In region II the water molecules start entering the crack. Here, *h*_0_ is greater than ≈5 Å and the slope of the sheet increases with *h*_0_, as can be seen in Fig. 2b. In region III, the sheet near the free edge is nearly vertical.

In the simulations, the fluid is in contact with the solid at all times for *v* < 50 m s^−1^: the fluid molecules penetrate the crack until there is enough spacing between the carbon atoms to accommodate a fluid molecule, *i.e.*, until a threshold sheet height of ≈5 Å. This motion is driven by pressure as the Péclet number (Pe) is larger than 1 for *v* > 10 m s^−1^ and the fluid is practically incompressible (the Mach number for *v* = 100 m s^−1^ is 
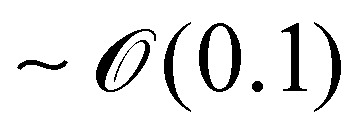
). Here Pe ≈ *v*ℓ/*D*_w_, where *D*_w_ ≈ 2.3 × 10^−9^ m^2^ s^−1^ is the diffusion coefficient of water^55^ and ℓ ≈ 3 Å corresponds to one molecular diameter.

To interpret the results in Fig. 2, we build a continuum model inspired by the MD system. The model complements the MD results by analysing the balance of the adhesive, bending, and viscous forces for rate-dependent peeling.

### 2.2 A continuum model

**Fig. 3:**
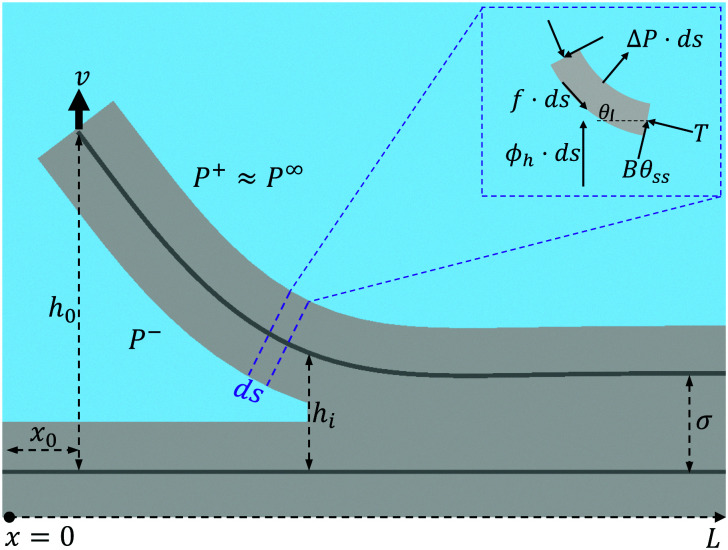
Schematic of the mathematical model. The deformable sheet is being pulled with a constant velocity *v* at the left edge. The blue shaded region represents the fluid of viscosity *μ*.

We consider a continuum model for the peeling of an elastic sheet from a stationary rigid surface. The sheet has length *L*, thickness *d*, and bending stiffness *B*. The deformable sheet is bound to the stationary surface by an adhesion force related to an adhesion potential *ϕ*. The left edge of the sheet is pulled upwards with an assigned velocity *v*, while being allowed to move freely in the horizontal direction. This latter condition is enforced by setting the horizontal components of bending and tensile stresses at the edge to be equal (Fig. 3). We assume that the sheet is inextensible. Therefore, while moving upwards the edge moves in the e⃑_*x*_ direction so that the inextensibility condition is satisfied at all times. An incompressible fluid of viscosity *μ* fills the gap until a minimal threshold gap height is reached (see below). Through its motion, the fluid exerts a tangential hydrodynamic traction *f* on the deformable sheet, and determines a pressure difference Δ*P* between the bottom and top surface of the sheet.

The shape of the sheet is described by a function *h*(*s*, *t*). When mapping to the MD simulations, *h* is the vertical distance between the centers of the atoms composing the deformable and stationary graphene sheets. Here *s* is the curvilinear coordinate line along the top sheet and *t* is the time. The corresponding Cartesian coordinates are *x*(*s*, *t*), *h*(*s*, *t*). Assuming that the sheet is inextensible, the angle of inclination of a point on the top sheet with the *x*-axis, *θ*(*s*, *t*), can be linked to *h*(*s*, *t*) and *x*(*s*, *t*) *via*

1 *h*_*s*_ = sin *θ*, *x*_*s*_ = cos *θ*,

where ()_*s*_ represents the derivative with respect to *s*.

The equations of equilibrium governing the shape of the deformable sheet can be obtained from a force balance on an element d*s* (Fig. 3),^56^ yielding

2 *Bθ*_*sss*_ + *Tθ*_*s*_ − *ϕ*_*h*_cos *θ* = Δ*P*,

and

3 *Bθ*_*ss*_*θ*_*s*_ − *T*_*s*_ + *ϕ*_*h*_sin *θ* = *f*.

Here *T* is the axial tension in the sheet, *ϕ*_*h*_ is the vertical adhesion force per unit area, Δ*P* = *P*^−^ − *P*^+^ is the difference between the normal forces per unit area exerted by the fluid below and above the sheet, and *f* is the corresponding tangential force per unit area (directed towards the crack tip). Without loss of generality, the pressure *P*^+^ is assumed to be zero. When the sheet is pulled upwards, the fluid exerts a downward hydrodynamic tension on the sheet, hence Δ*P*<0. The potential *ϕ*(*h*) models the adhesion of the deformable graphene layer with the stationary layer. The potential *ϕ*(*h*) is taken as a standard 4-10 Lennard-Jones potential between two thin plates, consisting of an attractive term and a repulsion term:^57–59^
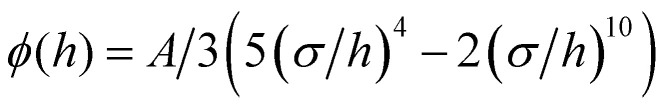
. Here *A* is the depth of the potential well and *σ* is the inter-layer equilibrium separation. Following previous work on graphene-graphene interactions,^58^ we set *σ* = 3.4 Å, a value that is also consistent with our MD simulations.

For given Δ*P* and *f*, eqn (1)–(3) are solved to find *θ*, *θ*_*s*_, *θ*_*ss*_, *T* and *h*. Five boundary conditions are required: at the right edge *s* = *L*, *θ*_*s*_ = *θ*_*ss*_ = 0 (zero moment and zero vertical force);^60^ at the left edge *s* = 0, *θ*_*s*_ = 0 (zero moment), *h*_*t*_ = *v* (kinematic condition) and [*Bθ*_*ss*_sin *θ* − *T*sin *θ*]_*s*=0_ = 0 (zero horizontal force). The upwards force acting on the left edge is

4 *F* = [*Bθ*_*ss*_cos *θ* + *T*sin *θ*]_*s*=0_

The adhesive and viscous contributions to *F* are calculated as5
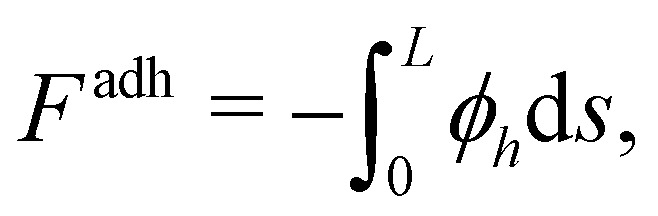
6
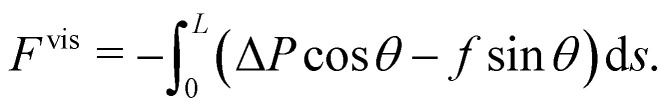


The finite van der Waals radius of carbon atoms makes the effective channel height available for the fluid molecules smaller than the channel height *h* defined from the center of the carbon atoms.^34,61,62^ Accordingly, hydrodynamic boundary conditions are applied at a distance *d*/2 from the center of each sheet. The effective height of the nanochannel is thus h* = *h* − *d*, with *d* = 2*σ*_C_ ≃ 3.4 Å where *σ*_C_ denotes the van der Waals radius of a carbon atom. The finite range of the carbon–water interaction leads to a threshold for *h* below which no fluid molecule fits in the nanochannel as discussed in Section 2.1. We choose this threshold height, *h*_*i*_ = 5 Å for water–graphene,^51^ a value consistent with observation from MD profiles (Fig. 2b).

The threshold sheet height results into an interface position *s*_*i*_ beyond which there is no fluid (Fig. 3). Therefore we set Δ*P* = 0 for *s* > *s*_*i*_. Due to the fluid flow, Δ*P* ≠ 0 left of this interface. Thus, there is a integrable discontinuity in the pressure across the interface at *s*_*i*_.^63^ The physics of this moving interface resembles that of the ‘dry cracking’ problem analysed by Lister *et al.* (2019), but is different from that of the ‘fluid lag’ concept,^64,65^ which requires a vapour region ahead of the interface. To account for the pressure discontinuity, we divide the *s*-axis into two domains, to the left and to the right of the interface *s*_*i*_. The solutions for the two domains are coupled to each other by enforcing continuity of *θ*, *κ*, *κ*_*s*_, *T* and *h* across *s*_*i*_.^60^

Applying mass conservation and accounting for incompressibility we obtain7
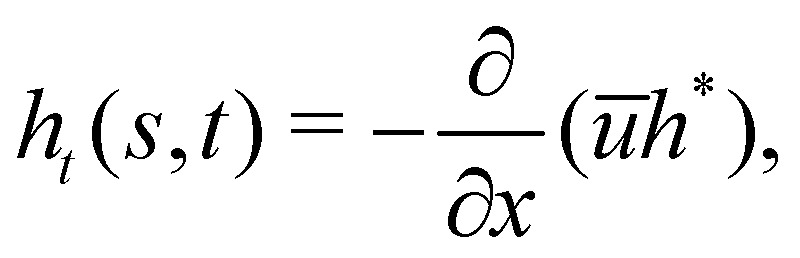
where *ū* is the height-averaged fluid velocity. Assuming that the liquid front position moves with a velocity equal to the average fluid velocity at that point, we arrive at the kinematic condition^63^8
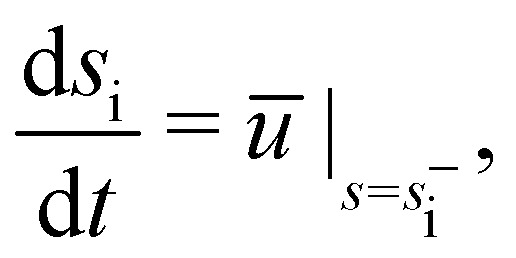
where the velocity is evaluated at positions just to the left of *s*_*i*_.

The eqn (1)–(3), (7), (8) are solved together with the corresponding boundary conditions using the Boundary Value Problem (BVP) solver of MATLAB.^66^ As initial condition, we use the stationary solution corresponding to a small assigned edge displacement *h*(*s* = 0) = *h*_*i*_. The initial shape of the sheet is obtained by running the iterative solver with *v* = 0 starting from the assigned shape9
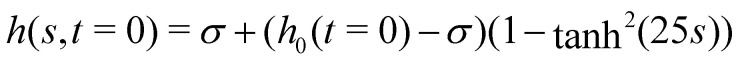
until convergence. The regions to the left and to right of *s*_*i*_ are discretized with two uniform meshes. We assume that the interface always moves in the positive *x*-direction as the sheet is peeled. Therefore, at each step of the iteration, the interface is moved from the previous position by a small amount equal to the grid spacing by using an implicit time-marching method. We repeat the above steps until *F* reaches a constant value. For the range of parameters we consider, this usually happens when the edge slope reaches π/2.

### 2.3 Comparison MD-continuum in the quasi-steady case

In this section, we consider quasi-steady peeling where viscous forces are absent (Δ*P* = *f* = 0) and therefore the forces on the sheet are rate independent. In the following we focus particularly on the prediction of the quasi-steady plateau force *F*^0^_plt_ and the maximum force *F*^0^_max_ obtained by solving eqn (1)–(3). The computed values of the force are compared with MD data to calibrate values of *A* and *B*.

In the limit *h*_0_ ≫ *σ*, the left edge has quasivertical orientation (*θ* (*s* = 0) ≈ π/2), the curvature of the sheet near the crack tip is approximately independent of *h*_0_^6^ and the bending energy is constant with respect to *h*_0_. The energy balance thus involves only the external work done by *F*^0^ and the adhesion work: ^6,14,67^

10 *F*^0^_plt_ ≈ *A*.

**Fig. 4:**
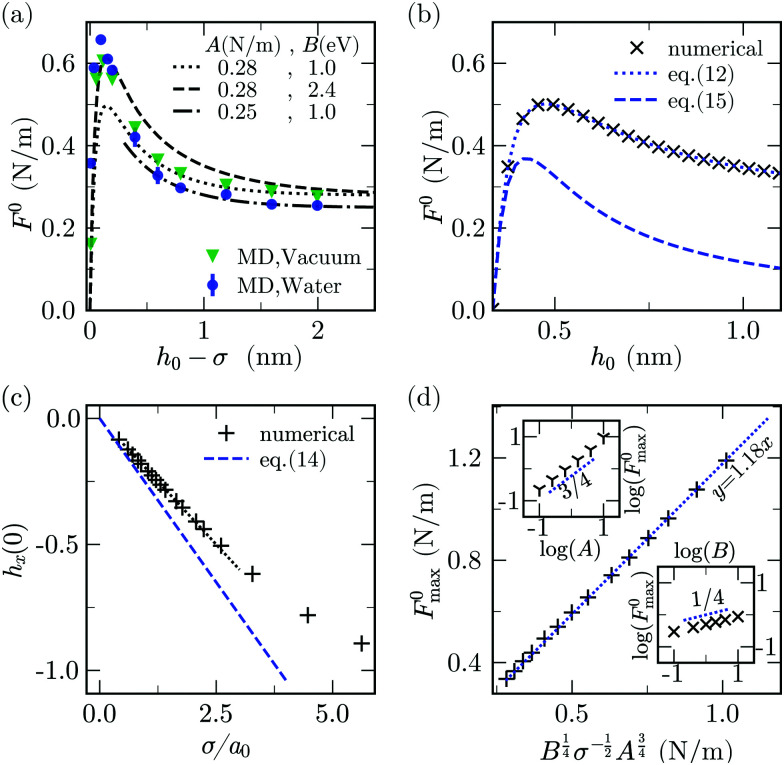
Quasi-steady peeling. (a) Dependence of *F*^0^ on *h*_0_, comparing the 1D continuum model (lines) and the MD data (solid symbols). The plot shows continuum simulations for 3 combinations of adhesion energy (*A*) and bending rigidity (*B*) and, MD simulations in vacuum and water as solvent. (b) *F*^0^*vs. h*_0_ calculated from eqn (12) and (15) compared with numerical solution for *A* = 0.28 N m^−1^ and *B* = 1 eV. (c) Dependence of *h*_*x*_(0) on 1/*a*_0_ for *h*_0_ = 1.26*σ* calculated numerically and from eqn (14). (d) *F*^0^_max_*vs.*
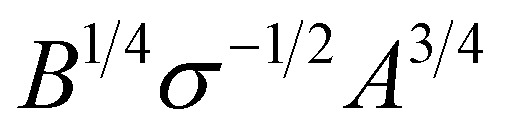
 and the dotted line is the plot for *y* = 1.18*x*. The top and bottom insets show the dependence of *F*^0^_max_ (N m^−1^) on *A* (N m^−1^) and *B* (eV) in log–log scale.

By calculating the work of adhesion required to separate two parallel sheets in vacuum using MD^25^ we obtain *A* ≃ 0.29 N m^−1^, a value close to the force plateau in vacuum ≃0.28 N m^−1^ (Fig. 4a).

To obtain an approximate analytical solution for *F*^0^_max_, we exploit the fact that the maximum value of the force is attained when slope of the sheet is small. For small slopes, the shape of the deformable sheet is governed by:^4,5,68^

11 *Bh*_*xxxx*_ − *ϕ*_*h*_ = Δ*P*.

The edge force *F*^0^ ≃ *Bh*_*xxx*_ can be obtained by multiplying eqn (11) by *h*_*x*_, integrating with respect to *x* and then using the boundary condition *κ*(0) = 0. The same result can be obtained by using the principle of virtual work.^69^ For Δ*P* = 0, we get12
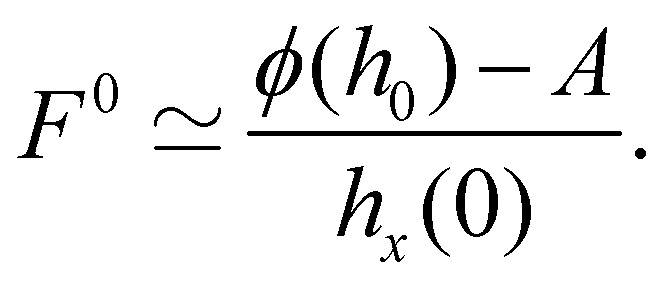
Inserting values of *h*_*x*_(0) in the above equation from numerical resolution of the continuum model, we get an excellent agreement with the complete numerical solution of *F*^0^ (Fig. 4b). We now express *h*_*x*_(0) as a function of *h*_0_, so that we can maximise *F*^0^ with respect to *h*_0_ to obtain *F*^0^_max_. Linearising *ϕ*_*h*_ in eqn (11) about the equilibrium separation gives13
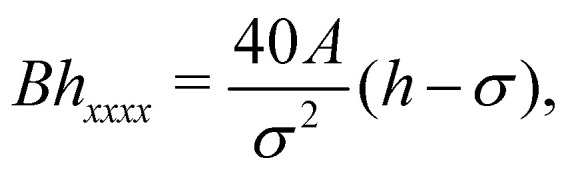
which has the solution *h* − *σ* = (*h*_0_ − *σ*) *e*^−*x*/*a*^_0_cos(*x*/*a*_0_), where 
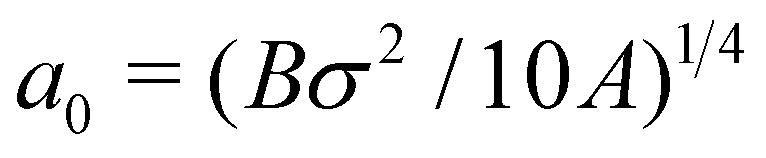
 is the cohesion length. Thus14
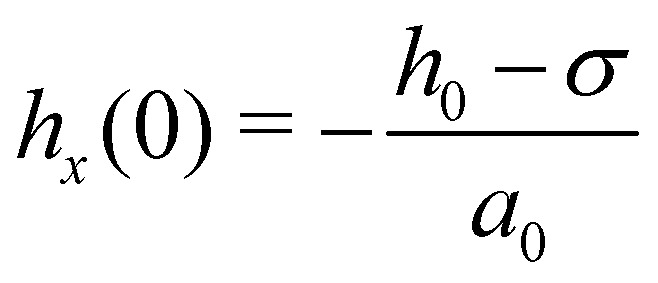
Plugging this relation in eqn (12) yields15
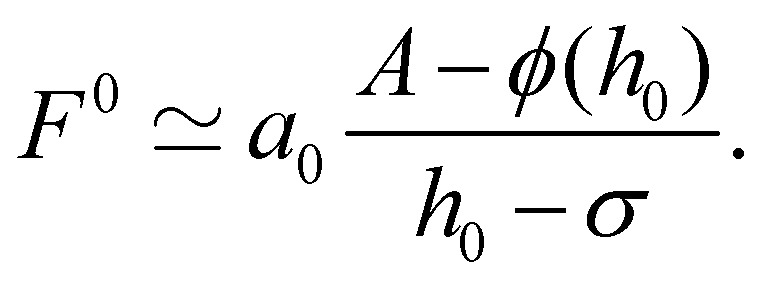
Using the expression for *ϕ*(*h*) (in Section 3), the above equation gives the same trend as the complete numerical solution (Fig. 4b) and has a fair agreement for the value of *h*_0_ for which *F*^0^ is maximum (*h*_0_ ≃ 1.26*σ*). For *σ*/*a*_0_ < 2, a condition which often holds in the context of thin film peeling experiments, the numerical solution confirms the 1/*a* dependency of *h*_*x*_(0) in eqn (14) at *h*_0_ = 1.26*σ* (see Fig. 4c). Maximizing *F*^0^ with respect to *h*_0_ gives16
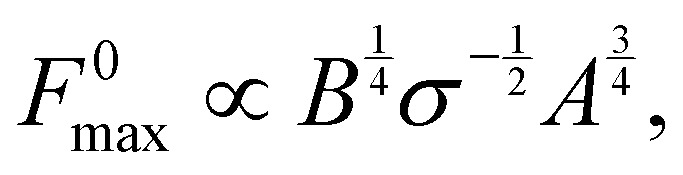
where the constant of proportionality is 
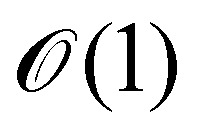
. We confirm the power-law relation of *A* and *B* with *F*^0^_max_ in eqn (16) by numerically computing *F*^0^_max_ for *A* in range [0.1,10] N m^−1^ (keeping fixed *B* = 1 eV and *σ* = 0.34 nm) and for *B* in range [0.1,10] eV (keeping fixed *A* = 0.28 N m^−1^ and *σ* = 0.34 nm) (Fig. 4d insets). As a further confirmation, *F*^0^_max_ is computed by varying *σ* in range [0.1,0.5] nm and *A*, *B* in the aforementioned range. Expressing *F*^0^_max_ as a function of 
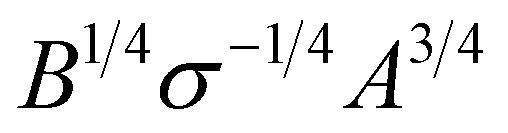
, the data collapses onto a straight line (Fig. 4d). We estimate the proportionality constant to be 1.18, close to the value of 0.88 obtained by exact minimisation of the small-slope expression. In summary, the maximum force is given approximately by17
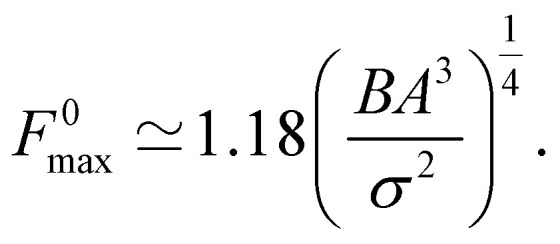
Ref. 27,70 showed that for small opening angle, 
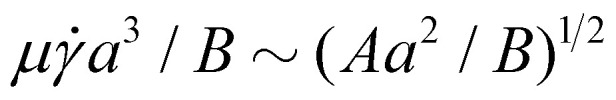
, where *γ̇* is the critical shear rate for exfoliation. Using *F*^0^_max_∼ *μγ̇ a* and *a*∼ *a*_*o*_, we arrive at the same scaling as in eqn17. This suggests that for *a*_*o*_ > 0.34*σ*, which turns out to be the case for the parameters *B*, *A* and *σ* characterising our simulations, the force *F*^0^_max_ is larger than *F*^0^_plateau_ by a factor 
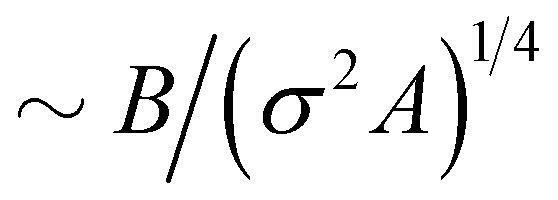
. In peeling of macroscopic elastic films *B* is much higher than in graphene.^4,71–74^ Our predictions for the maximum and plateau forces are based on a continuum framework, and therefore hold for macroscopic systems provided that *a*_0_ ≫ *σ*.

The values of *F*^0^_max_ and *F*^0^_plateau_ depend on *A* and *B*. We can therefore use the quasi-static estimates of peeling force to calibrate these two parameters. Using eqn (10) for peeling in vacuum we obtain *A* ≃ 0.28 N m^−1^, a value that is very close to the graphene-graphene adhesion energy reported in previous MD simulations and experiments.^75,76^ For peeling in water, we obtain *A* ≃ 0.25 N m^−1^, comparable to the value in vacuum.^24,25^ Previous experiments and MD simulations estimated the adhesion energy of graphene in water to be of the same order magnitude as found here.^3,25,77,78^ Knowing the adhesion energy, eqn (17) can be used to calculate the bending stiffness. The value we obtain is *B* = 2.4 eV. However for *h*_0_ > 0.8 nm fitting the continuum peeling force with MD using BFGS method^79^ yields *B* = 1.0 eV. This suggests that the bending stiffness varies between 1.0–2.4 eV depending on *h*_0_. Previous works have also reported a variation of bending rigidity in a similar range, depending on the extent of deformation of the sheet.^80,81^

**Fig. 5:**
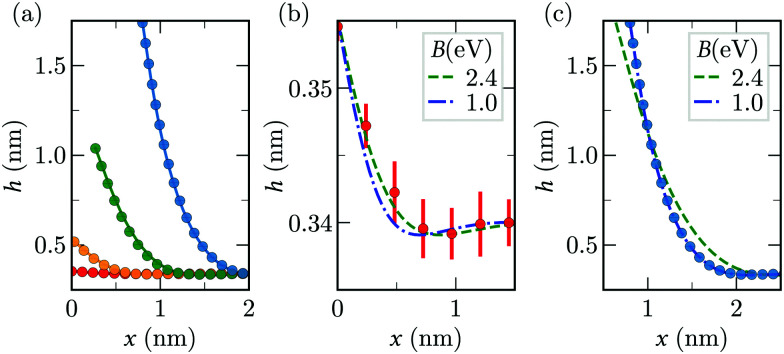
Comparison of quasi-steady sheet profiles from the continuum model (lines) and MD simulations (disks) in vacuum. (a) Sheet profiles for 4 different values of *h*_0_, (0.355, 0.52, 1.04, 1.75) nm. The parameters *A* = 0.28 N m^−1^ and *B* = 1 eV for the continuum profiles. (b and c) The MD profiles for *h*_0_ = 0.355 nm and *h*_0_ = 1.75 nm respectively, compared with continuum profiles for two values of *B*. Here, *A* = 0.28 N m^−1^.

We compare sheet profiles in the continuum model with those obtained from MD. The quasi-static evolution of sheet shape in MD shows good agreement with that obtained by solving eqn (2) and (3) with the fitted MD values of *B* and *A* for vacuum (Fig. 5a). The sheet profiles in Fig. 5b and c confirm our observation on the variance of *B* on *h*_0_: *B* = 2.4 eV shows a slightly better fit for *h*_0_ = 0.355 nm while *B* = 1.0 eV is a considerably better fit for *h*_0_ = 1.75 nm. In the cohesive region, the oscillatory pattern visible in Fig. 5b is a consequence of the competition between the bending and adhesive forces.^82^ In the following we use *B* = 1.0 eV and *A* = 0.25 N m^−1^ when discussing the values of *F* for graphene–water system.

### 2.4 Analysis of the velocity-dependent case

In this section, we analyze the effect of rate-dependent peeling on the sheet's shape and peeling force. To get insights on the dependence of the shape of the sheet on the peeling velocity, we first write eqn (2) and (3) in dimensionless form. With the horizontal length of the channel scaling with 
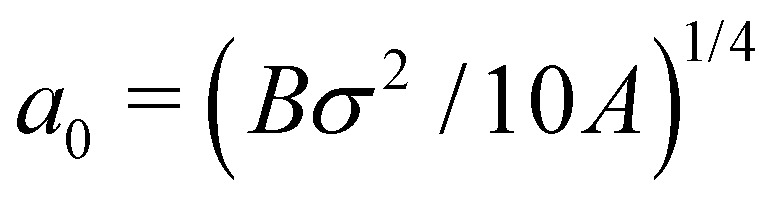
 and estimating the characteristic curvature as 
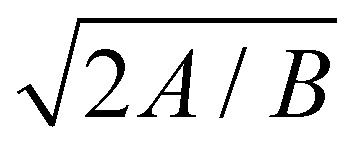
,^56,83,84^ the characteristic slope near the crack tip is 
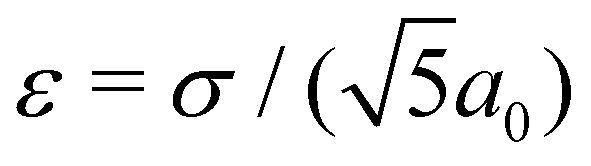
. We rewrite eqn (2) and (3) using the following dimensionless variables (represented with hat symbols):18
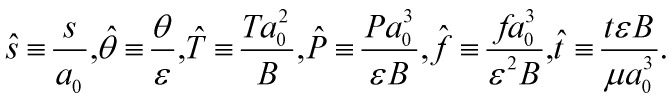
The dimensionless governing equations are19
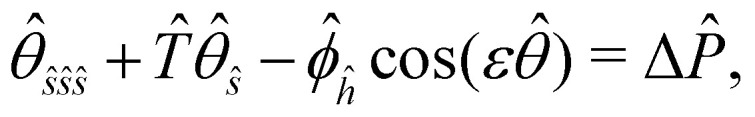
20
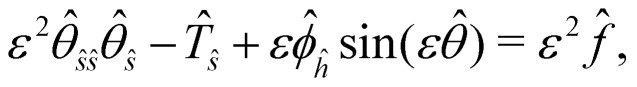
where 
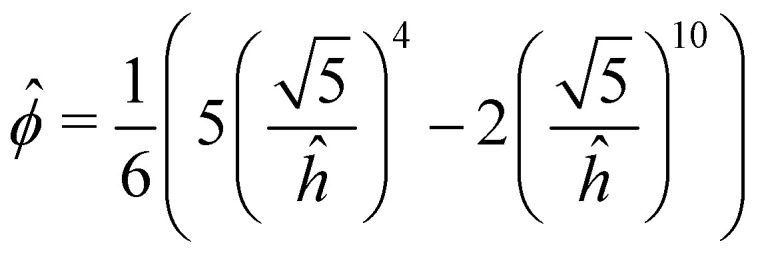
 and *ĥ* = *h*/(*ε a*_0_). The dimensionless sheet thickness is *d̂* = *d*/(*εa*_0_) and the dimensionless pulling velocity is *v̂* = *μv*/(2*A*) (With the normalisation eqn (18), *P̂* and *f̂* are the ratios of the pressure and friction terms to the first terms in (19) and (20), respectively. For *ε* ≪ 1, the small slope limit of the balance equations is recovered.). Characteristic values from our MD simulations are *μ* = 10^−3^ Pa s and *A* ∼ 0.1 N m^−1^, hence *v̂* turns out to be in the range [0.005–0.5] for *v* ∈ [1–100] m s^−1^. In eqn (19) and (20), Δ*P̂* is an increasing function of *v̂*, so there is a lower range of value of *v̂* for which Δ*P̂* ≪ 1 and *f̂* ≪ 1, *i.e.* the quasi-static case.

**Fig. 6:**
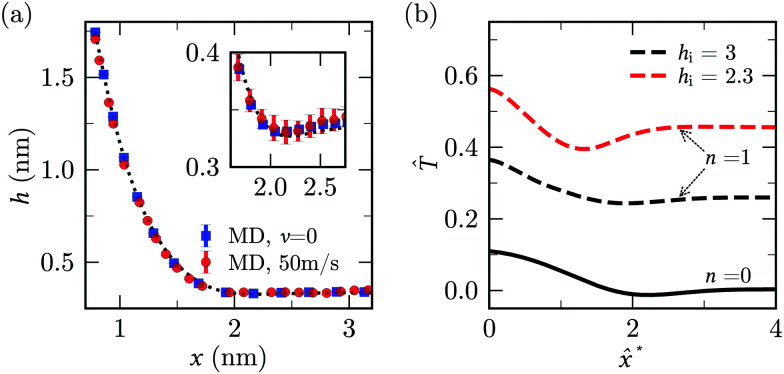
Rate dependence of sheet profile and tension. (a) Sheet profiles from MD for *h*_0_ = 1.75 nm at *v* = 0 (squares) and *v* = 50 m s^−1^ (disks). Dotted line shows solution of continuum model (*A* = 0.25 N m^−1^, *B* = 1.0 eV) with Δ*P* = *f* = 0. (b) Non-dimensional tension along the sheet for the pressure profile in terms of *n* defined in eqn (21) with two values of *ĥ*_*i*_ and *ĥ*_0_ = 4.

Examining the effect of *v* on the sheet shape extracted from MD (Fig. 6a), we noticed that the profile of the sheet is practically independent of *v* for the range of velocities we considered. To interpret this observation, we solve the coupled eqn (19) and (20) with a simple prescription for Δ*P̂* in which Δ*P̂* = −*n* for *ĥ* greater than the interface height *ĥ*_*i*_ and zero otherwise:21
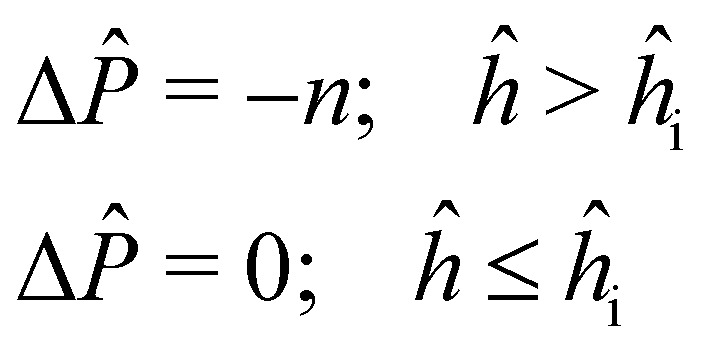
Here we fix *ε* = 0.5, *f̂* = 0 and *n* = 0 or 1. If the slope is not negligible, an increase in peeling force with Δ*P̂* must be accompanied by an increase in tension at the left boundary (eqn (4)). From eqn (20), in the case when *θ̂* and its first two derivatives are independent of Δ*P̂*, *T̂*_*ŝ*_ remains nearly constant, and therefore the tension curve must uniformly shift upwards with the increase in tension at the boundary. Considering that for *n* = 1, there is a significant increase in *F̂*, we find that *T̂* indeed shifts upward with *n* for *ĥ*_*i*_ = 3 (Fig. 6b). However, the increase for *ĥ*_*i*_ = 2.3 is notably non-uniform implying a significant change in shape. A smaller value of *ĥ*_*i*_ means that the fluid wets further inside the sheet at the peeling front. Therefore, the unvarying shape of the sheet with *v* can be explained by the absence of fluid in the curved part of the sheet near the front (see Fig. 2a).

The fact that the shape of the sheet is approximately independent of the peeling velocity even in presence of water means that we can estimate the bending and adhesive forces from the quasi-static results, and use these estimates when the velocity is not small. As a corollary, we can decompose unambiguously the peeling force *F* into a non-dissipative contribution, given by eqn (10) for large angles and eqn (12) for small angles, and a velocity-dependent viscous contribution.

**Fig. 7:**
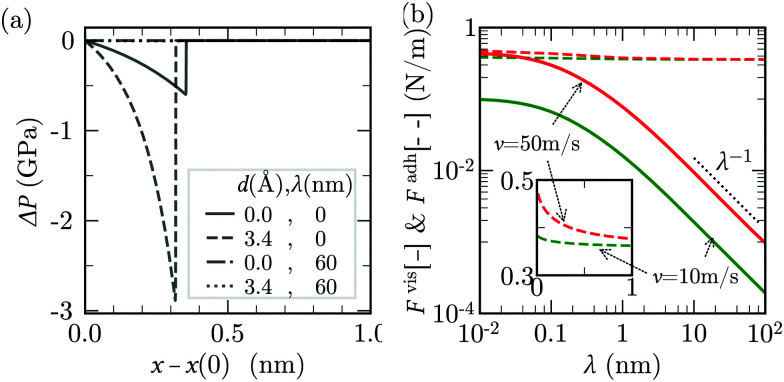
Results of lubrication model described by eqn (22) and (23). (a) Comparison of lubrication pressure profile along the sheet (*h*_0_ = 0.8 nm) from the continuum model for different combinations of *λ* and *d*. (b) *F*^vis^ (solid lines) and *F*^adh^ (dashed lines) as a function of *λ* using eqn (22). The peeling velocities, *v* = 50 m s^−1^ (red) and *v* = 10 m s^−1^ (green), *h*_0_ = 0.8 nm and *B*, *A*, *σ*, *d* are for graphene-water system. The inset is an enlarged version of the same plot showing the difference in *F*^adh^ values.

As customary in elasto-hydrodynamic problems, we investigate the relation between pressure-drop and axial velocity in the lubrication limit of small slopes.^5,85–87^ From the continuum model, we analyse the velocity dependent case for small deflections (*i.e.*, *h*_0_ < 1 nm) as the 1-D approximations for pressure are valid only for small slopes. In this limit, for stationary channels presenting Navier-slip boundary conditions at the walls, the depth-averaged fluid velocity is related to the pressure gradient *via*^88^22
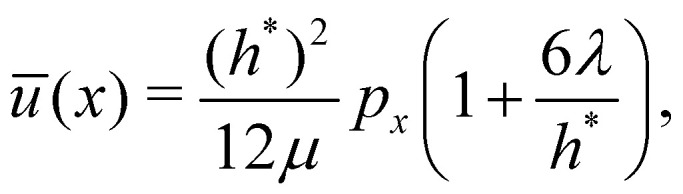
which recovers the no-slip expression for *λ* ≪ h*/6. Here the height of the channel is taken to be h* = *h* − *d*, to account for the thickness of the sheet (see Section 2.2). Eqn (22) is in the form used, *e.g.*, to model the flow of thin liquid films in the weak-slip regime.^89–91^ Momentum balance in the flow direction gives23
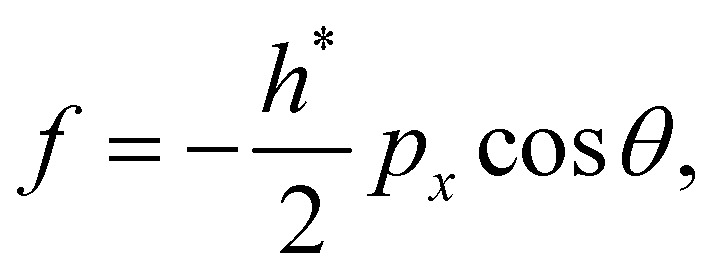
where *f* = *μ*(d*u⃑*/d*y*)·*n⃑* is the tangential friction and *n⃑* is the unit vector normal to the boundary. We solve eqn (2) and (3) with the prescription of pressure and friction described above^4,5^ and *p*|_*s*=0_ = 0 (Fig. 7). From the shape of the sheet, we calculate the edge force using eqn (4).

If the slip length was zero, the crack propagation would result into a diverging viscous stress near the crack tip,^85,92^ as in the moving contact line problem.^93,94^ In theory of moving contact line,^93,95^ introduction of slip regularises the stress divergence. The slip lengths are comparatively large in our MD simulations (*λ* ≃ 60 nm ≫ *a*_0_ ≃ 0.3 nm)^38^ and significantly reduce the magnitude of the viscous stress near the crack tip (Fig. 7a). The choice of *d* has no noticeable effect on lubrication pressure as *λ* ≫ *h*− *d*.

For large slip lengths (6*λ*/h* ≫ 1), the viscous component of *F* due to lubrication is much smaller than the adhesive component even at relatively large peeling velocities (Fig. 7b). As an instance, for *λ* = 10 nm and *v* = 50 m s^−1^ the viscous component *F*^vis^ is about one order of magnitude smaller than *F*^adh^. If the shape of the sheet is invariant with respect to *v*, the lubrication pressure for *λ* ≫ h* should scale, according to (22), approximately as24
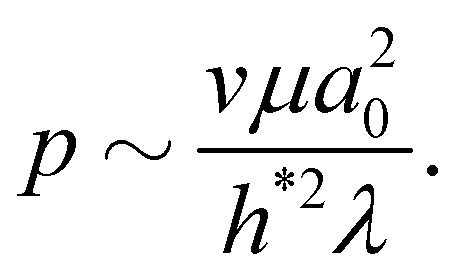
Here, we use the continuity equation for small slopes, which yields 
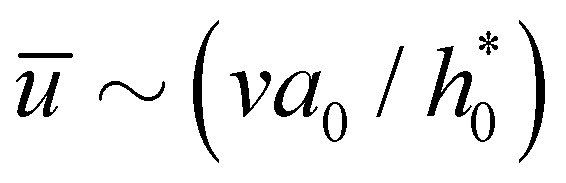
. Therefore, *F*_vis_ ∝ 1/*λ* for sufficiently large *λ*, which our continuum simulation confirms (Fig. 7b). The criterion to neglect viscous stresses due to lubrication (*P̂* ≪ 1) yields the following “large-slip” condition:25
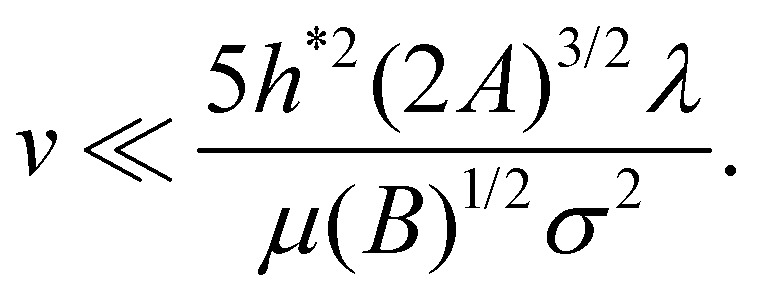


Using eqn (25), we find that the viscous component of *F* arising from lubrication can be neglected for *v* ≪ 10^5^ m s^−1^ in our MD simulation. Thus, lubrication forces cannot explain the dependence of peeling force on velocity seen in the MD results of Fig. 2d.

**Fig. 8:**
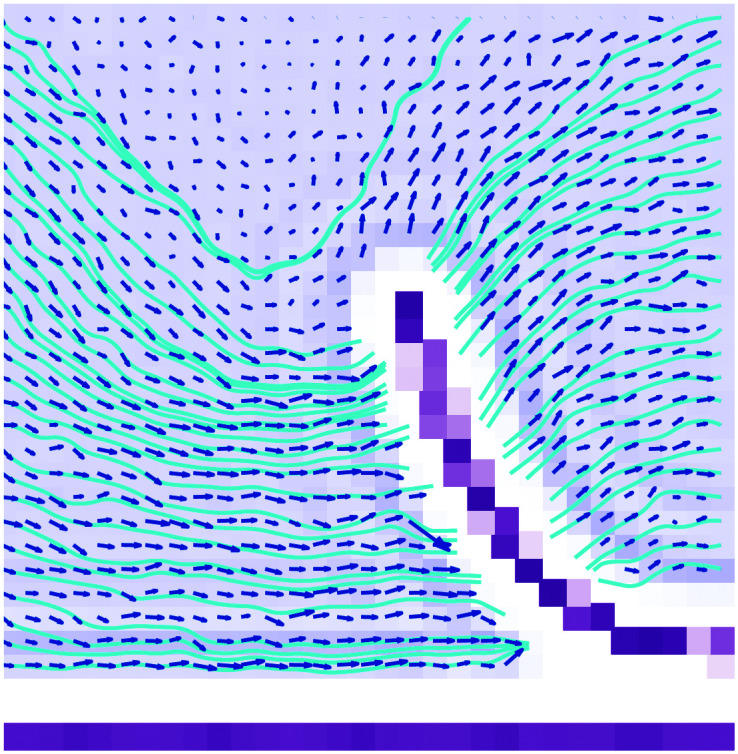
Fluid velocity vector field computed from molecular dynamics simulation. The blue lines denote velocity streamlines. The data is averaged over 8 simulations at *v* = 50 m s^−1^.

In the MD simulations of confined liquid in nano-channels,^96–98^ a large hydrodynamic slippage is known to generate a more uniform flow profile (the usual parabolic flow expected for pressured driven flow in no-slip channels reduces to a uniform flow as the slip length increases^29,99^). Similarly, in the problem considered in the current paper the effect of large slip is to make the velocity field near the crack tip approximately uniform (Fig. 8). The streamlines (in the frame of reference of the stationary layer) near the crack-tip are parallel to the lower boundary. The velocity field does satisfy both the no-penetration and the tangential slip boundary conditions at the liquid–solid boundaries. A small e⃑_*z*_ component of the fluid velocity can only be seen close-to the edge, above the sheet and in front of the entrance of the flap.

**Fig. 9:**
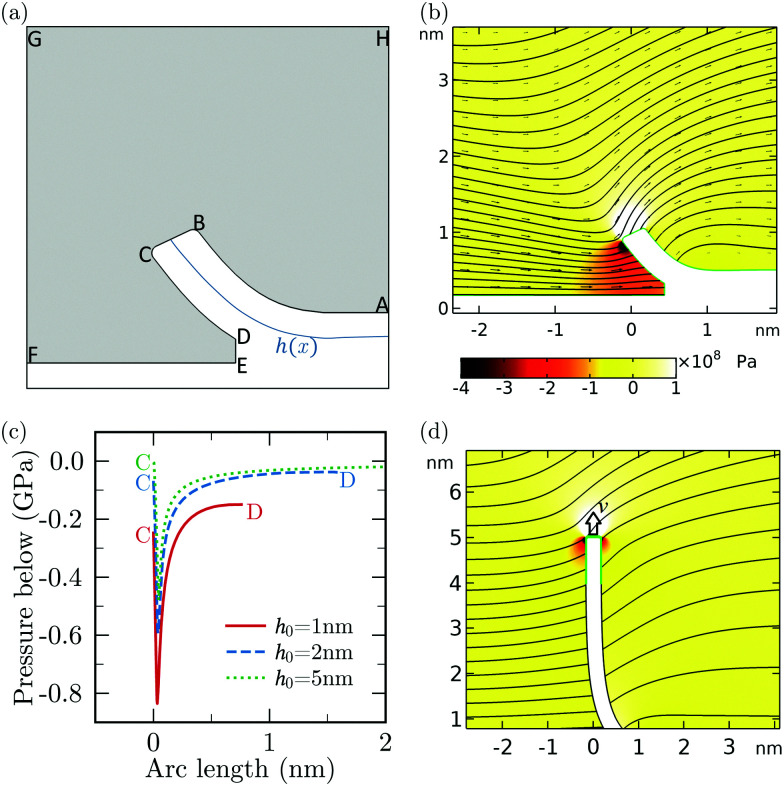
Analysis of COMSOL simulations of the dynamically peeled sheet. (a) Schematic of the simulation for *h*_0_ = 1 nm. The region with fluid is shaded with grey. The reservoir boundaries are shown not to scale. (b) Representative pressure and velocity field. (c) Absolute pressure just below the sheet (along CD) extracted from the simulation for different peeling heights for *v* = 50 m s^−1^. (d) Simulation schematic illustrating the configuration of sheet for *h*_0_ = 5 nm and the corresponding velocity streamlines. The surface near the edge of the sheet (corresponding to *y* > 4 nm) is highlighted in green.

Calculating pressure, viscous shear and normal stresses from MD is challenging due to thermal noise and the difficulty of applying volume-average to smallgap regions. Therefore, we have performed continuum simulations using a finite element software (COMSOL) of a peeled sheet of finite thickness for 4 values of *h*_0_ moving at different peeling velocities (Fig. 9a). In these simulations, we solve the incompressible Stokes equations

26 ∇*p* = *μ*∇^2^**u**; ∇·**u** = 0

where *p* is the pressure field and **u** the velocity field, with free slip boundary condition at all surfaces, corresponding to *λ* = ∞. The boundaries, ABCD in Fig. 9a, are prescribed based on the solution of eqn (2) and (3) for Δ*P* = *f* = 0 (see Fig. 6a). The boundaries are placed at a distance *d*/2 from the centre-line positions of the moving and stationary sheets, given by *y* = *h*(*x*) and *y* = 0 respectively. To avoid singularity, point B and C are replaced by rounded corner of radius *r* = 0.5 Å. To avoid finite size effect due to the reservoir, the height (FG) and width (GH) of the computational domain is chosen to be much larger than any other dimension. The interface (segment DE in Fig. 9a) of height *h*_*i*_− *d*, located at position *s* = *s*_*i*_, moves with velocity *u*_*i*_ in the e⃑_*x*_ direction, where *u*_*i*_ = d*s*_*i*_/d*t* is the average fluid velocity at *s*_*i*_. The reservoir walls are modelled as zero pressure gradient outlets. We perform simulations for *h*_0_ = 1, 2, and 5 nm.

**Fig. 10:**
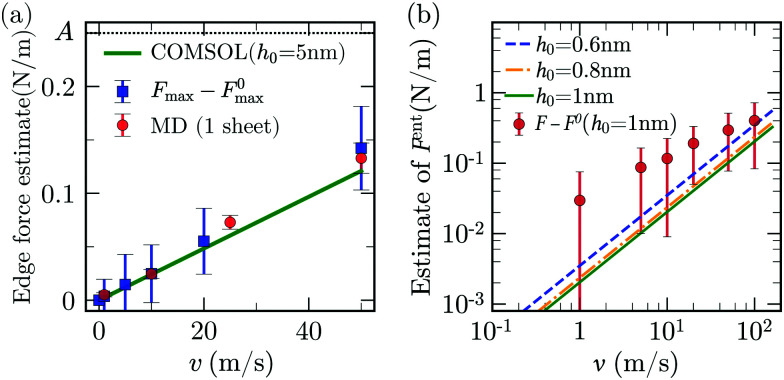
(a) Viscous resisting force for a single nanosheet moving upwards with velocity *v* (red disks); *F*_max_ − *F*^0^_max_ from MD (blue squares); integral of the y-component of the stress near the edge of the sheet from COMSOL integrated along the surface highlighted in green in Fig. 9d (line). Here, *A* = 0.25 N m^−1^ is the adhesion energy measured in water. (b) Estimate of *F*^ent^ from eqn (27) for *h*_0_ = 0.6, 0.8 and 1 nm. Red disks are *F* − *F*^0^, *i.e.*, the total increase in peeling force in MD at *h*_0_ = 1 nm.

Fig. 9b shows the fluid velocity vectors and the corresponding pressure distribution for *v* = 50 m s^−1^ and *h*_0_ = 1 nm. The pressure in between the gap is predominantly negative, with a large negative pressure in the region surrounding the edge. The absolute pressure along the arc CD has a sharp minimum near the edge and then increases smoothly as the crack tip is approached (Fig. 9c). A convective motion of fluid around the edge of the sheet is visible in Fig. 9b and 8. The integral effect of the viscous and pressure stresses associated to this convective motion give rise to a drag resistance, which we term “edge drag” force. To characterise the magnitude of this force, we have developed two independent estimates. One is based on the COMSOL simulations discussed previously. We consider the case of *h*_0_ = 5 nm shown in Fig. 9d for different values of *v* as the viscous effect of the edge can be isolated in this configuration. To estimate the viscous stress at the edge, we integrate the y-component of stress along the surface of the sheet above *y* = 4 nm (highlighted in green in Fig. 9d). The estimated viscous stress increases approximately linearly with *v* and is comparable in magnitude with *A* for *v* > 10 m s^−1^ (Fig. 10a). As a second estimate, using MD simulations we have computed the vertical force on a flat rigid vertical nanosheet of length ∼ 1 nm moving vertically with an assigned velocity *v*.‡‡As this sheet has two identical ends, the drag force at each end of the nanosheet (red circles in Fig. 10a) is calculated as half of the required pulling force. In both cases we get values which are very close, in magnitude and trend, to the value of *F*_max_ − *F*^0^_max_. Both estimates give values of the edge force comparable in order of magnitude to the quasi-steady adhesion forces for *v* > 10 m s^−1^. Therefore, the sharp minimum in (Fig. 9c) can be attributed to the motion of fluid displaced as the edge moves upward.

The “edge drag”' force does not capture all the viscous forces as it does not explain the increase in *F* − *F*^0^ occurring when fluid starts entering the crack (region II of Fig. 2c). The fact that the pressure does not suddenly increase to zero and saturates to a significant value for *h*_0_ = 1 nm (Fig. 9c) suggests that another source of pressure drop in our system can be due to the motion of fluid entering the gap between the sheets. To estimate this contribution, we refer to previous results on pressure-driven flow in two-dimensional channels which share similarities to the entrance flow below the sheet and near the edge in our configuration. Hasimoto (1958)^100^ studied analytically the Stokes-flow hydrodynamic resistance of an infinitely thin plate presenting a slot of height 2*h*. This configuration is relevant to our case when *h*_0_ is small and the channel walls are thus nearly parallel. The pressure drop along the channel was characterised in terms of the hydrodynamic resistance *R*_H_, *i.e.* the ratio of the magnitude of the pressure drop to the volumetric flow rate *Q* ∼ *ūh*. Hasimoto's solution gives27
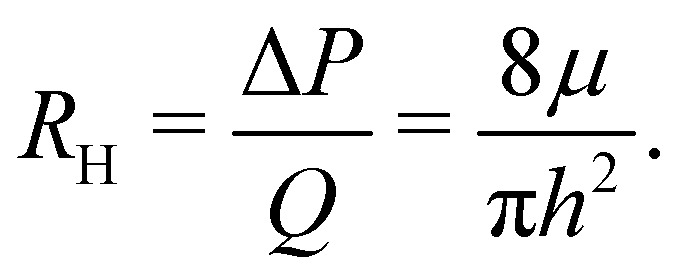
Hasimoto's formula is not an accurate representation of hydrodynamic resistance for smoothly-converging channels, so the accuracy of eqn (27) decreases for increasing *h*_0_. Numerical studies of pressure-driven flow in smoothly converging axis-symmetric entrances with fixed walls show that *R*_H_ depends on the ratio of *λ* to the minimal channel height *a*, the ratio of the channel length and *a*, as well as the specific shape of the channel.^101–104^ For completeness, a COMSOL analysis of the entrance resistance *R*_H_ for pressure-driven flow in smoothly-converging stationary 2D channels (as opposed to axi-symmetric) in the case of infinite slip length, for different values of the radius of curvature of the entrance, is presented in the ESI.† The analysis show that Hasimoto's solution gives values of *R*_H_ only 20% smaller than those provided by COMSOL when the radius of curvature of the entrance is comparable to, or smaller than, the minimum channel height (see Fig. S4 in ESI†). Therefore, we use eqn (27) as an approximation for small values of *h*_0_. We notice that a viscous resistance due to converging streamlines is, physically, akin to the resistance due to extensional viscous stresses described in the lubrication formulation of ref. 90, 105 and 106, except that in our case the gradients are large because the fluid layer is not slender.

We compute Δ*P*, appearing in eqn (2), using eqn (27) with the available channel height 
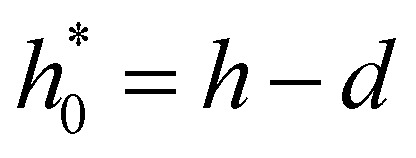
 in place of *h*, and evaluating *Q* from the average velocity defined in eqn (7). We calculate the component of the force associated with entrance flow, *F*^ent^, from the total force by removing the adhesive component (eqn (6)). We find that *F*^ent^ is comparable to, but smaller than, the value *F* − *F*^0^ extracted from MD at *h*_0_ = 1 nm (Fig. 10b). This suggest that entrance flow is an important contribution to the viscous resistance to the peeling front motion.

As *F*_max_ lies in region I of Fig. 2c, we infer that the viscous contribution to *F*_max_ must arise from the motion of the fluid mostly close to the edge, because there is practically no fluid in between the sheets in region I. The peeling force in region II & III will instead receive contributions from both the edge and the entrance resistance. Indeed, the difference between *F* − *F*^0^ and the theoretical values for *F*_ent_ in Fig. 10b is comparable to the viscous force due to the edge drag in Fig. 10a. So, the sum of these two contributions is close the total viscous resistance (not exactly equal, expectedly, due to the use of simplified models). Using the COMSOL estimate for the edge drag force and eqn (27) for the entrance pressure drop in our continuum model, we obtain an increase in *F* with *v* for *v* > 1 m s^−1^. This increase in *F* is qualitatively similar to that suggested by the MD data in Fig. 2c. To build an accurate model, one needs to separate the edge and entrance contributions to the viscous resistance in MD. However, a systematic way to separate these two contributions was unfortunately not found since both contributions are, to a first approximation, linear in the velocity and their magnitudes are also comparable.

**Fig. 11:**
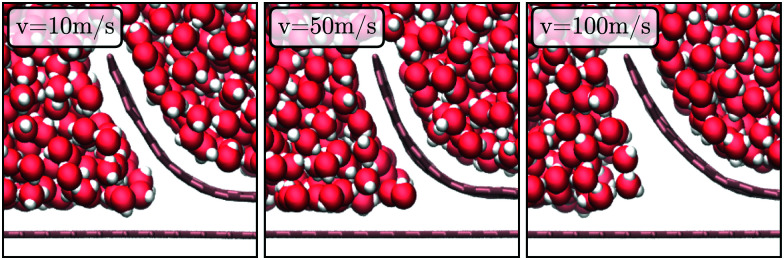
Snapshot of MD simulation near the crack tip at different peeling velocities for *h*_0_ = 1.6 nm.

The MD results in Fig. 10a and b show approximately a linear trend with *v*, except at *v* = 100 m s^−1^. For this particular value the MD data for the force is lower than expected by extrapolation of the force-velocity curve at smaller velocity. Fig. 11 illustrates that this velocity value corresponds to the formation of a vacuum pocket (cavitation) near the crack tip. (At this velocity value, capillary forces may play an important role.) Cavitation at large peeling velocity have been reported^10,107^ in the case of peeling of a scotch tape. In molecular dynamics, the threshold negative pressure for cavitation in pure water is reported to be ∼0.2 GPa,^108,109^ which is of the same order as the pressure near the crack measured from our COMSOL calculation.

### 2.5 Threshold velocity

In the initial stage of peeling, using eqn (13) the slope at the entrance is 
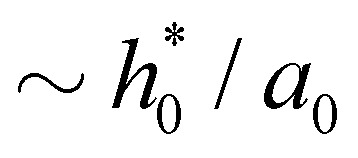
. Balancing the terms in the fluid continuity equation gives 
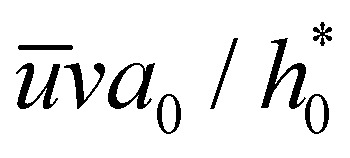
. Thus,28
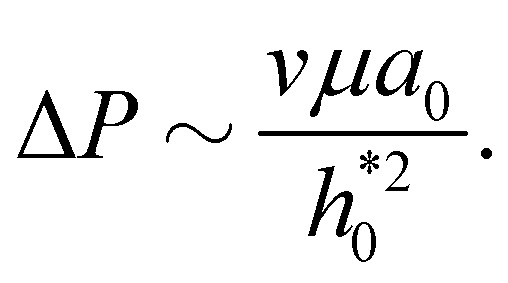
To obtain this expression we have here used the scaling 
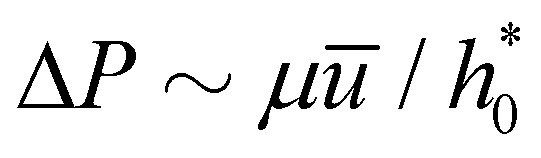
 suggested by Hasimoto's solution (27). Using a practical numerical threshold of 0.01 in place of ≪ 1, the condition Δ*P̂* ≪ 1 translates to a practical threshold of29
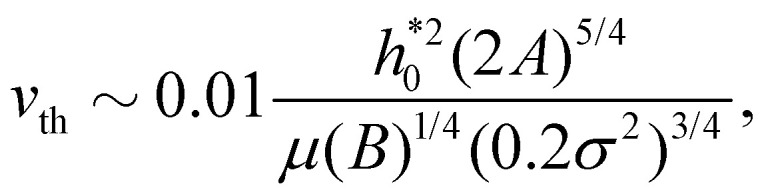
to be compared with the lubrication estimate (25). The entrance-flow estimate (29) gives *v*_th_ ∼ 1 m s^−1^ in our problem of peeling of mono-layer graphene in water.

**Fig. 12:**
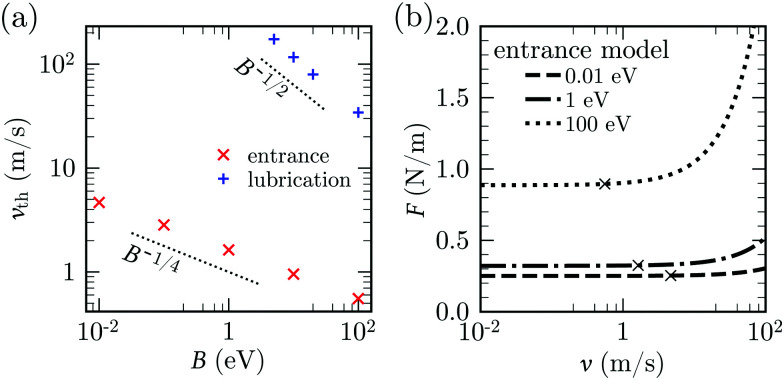
Threshold peeling velocity. (a) *v*_th_ for entrance model (cross symbol) and lubrication model with *λ* = 60 nm (plus symbol) as a function of *B*, keeping *A* = 0.25 N m^−1^, *σ* = *d* = 0.34 nm, *h*_0_ = 1 nm and *h*_*i*_ = 0.5 nm. (b) *F vs. v* for different values of *B* for entrance model, keeping other parameters same as (a). The value *v*_th_ (cross symbol) is defined as the point where *F* = 1.01*F*^0^.

The parametric dependence of the threshold velocity using the entrance pressure is different from the one obtained using the large-slip lubrication pressure. This is because the typical length-scale characterising the velocity gradients in large-slip lubrication is 
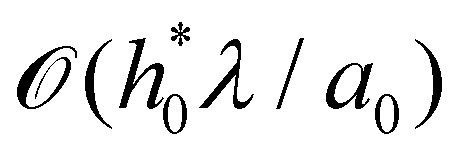
, while it is 
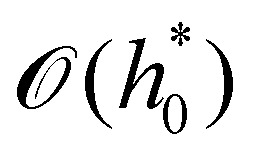
 in entrance flow. Fig. 12, obtained with the 1-D continuum model, confirms the ∼*B*^−1/2^ and ∼*B*^−1/4^ dependencies of the threshold velocity in the large-slip lubrication case and in the entrance flow case, respectively. In this test we define *v*_th_ as the velocity for which *F* increases by 1% with respect to the quasi-steady value. In the literature on silicon wafer bonding in air, the velocity of crack has been reported to have the same scaling in *B* and *A* as in eqn (29).^71^ An inverse 3/2 dependence with sheet thickness has also been previously reported.^110^

Eqn (29) predicts an increase in threshold velocity with adhesion energy. The adhesion force increases with *A* and the peeling velocity at which viscous forces start becoming comparable to adhesion forces also increases. The inverse dependence on *σ* can be explained similarly: for the same value of 
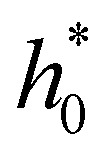
, adhesion forces increase with decreasing *σ*. Eqn (29) predicts a weak inverse dependence on bending rigidity. A more rigid sheet leads to smaller deformations, larger crack lengths and larger fluid velocity at the entrance. Hence the total viscous force on the top sheet is increased. This increment in viscous force must be larger than the increment in the quasi-steady peeling force (with *B*) at small slopes. Our continuum model indeed confirms that *F*, unlike *v*_th_, increases with *B* (Fig. 12b).

Comparing the scaling for lubrication pressure and entrance pressure (eqn (24) and eqn (28) respectively), we get the condition *λ*/*a*_0_ ≫ 1 to neglect the effect of the lubrication pressure in comparison with the entrance pressure for small peeling angles.

### 2.6 Comparison with the literature

For the static peeling, we define a horizontal length scale *a*_0_ from the balance between adhesive and bending stresses near the crack tip. Previous studies^28,111^ suggest that the stresses at a length 
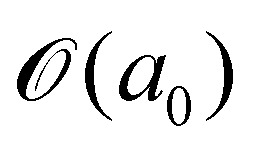
 near the peeling front control the shape of the peeled flap. In our case, *a*_0_ ∼ 0.3 nm, comparable to the size of one fluid molecule, and comparable to the length of the curved part of sheet near the peeling front. As fluid is absent in the curved part for *s* > *s*_*i*_, the shape of the peeling front in the neighbourhood of this region is approximately quasi-static, with characteristic outer curvature 
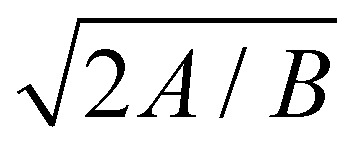
.^112^ Away from the front, the shape is practically linear owing to the zero moment condition at the edge. Correspondingly, the shape of the sheet is approximately independent of viscous effects. Our case is contrary to that of an elastic polymer sheet with a pre-wetting layer of liquid underneath, where the viscous effects near the peeling front control the sheet's shape.^1,4,63^

In previous studies on viscous peeling, the fluid is either present only inside the crack^1,60,63^ or confined within a blister;^4,28,68^ in these studies lubrication dissipation due to a Poiseuille flow in the gap was the primary source of viscous dissipation. In contrast, our configuration is completely immersed in the fluid. Therefore, viscous dissipation also comes from the motion of the edge relative to the surrounding fluid^27^ and suction of fluid from the outer “reservoir” as the opening angle increases.^101^ When the slip is large compared to the system's dimensions, these forms of dissipation dominate over the usual lubrication dissipation.

## 3 Summary and outlook

We have carried out MD simulations of peeling of a deformable graphene layer in the presence of liquid water. The MD data is rationalised by a 1-D continuum model based on equilibrium equations for large deflections of a non-linear *elastica*. These solid mechanics equations have been used to model, *e.g.*, inextensible elastic rods^113,114^ and fibres,^115^^116^ also for large deflections. Finite-element COMSOL simulations are also carried out to investigate certain flow features and to better characterise the hydrodynamic load terms present in MD. The effect of identified viscous stresses are estimated, for small deflections, using the 1-D model.

In the quasi-static regime, we have been able to capture with the 1-D model the dependence of the peeling force on the edge height *h*_0_, provided that two values were used for the bending rigidity, one for small deformations, and another for large deformations. This feature is likely due to the dependence of the mechanical response of the crystal structure of graphene to the tension applied to it.^117^ The quasi-static force profile as a function of *h*_0_ displays a maximum for intermediate values of *h*_0_, and then reaches a plateau. The plateau force in MD was found to scale linearly with the adhesion energy *A*, independent of the bending rigidity *B* of the sheet. The maximum quasi-steady peeling force instead was found to scale proportionally to *B*^1/4^ and *A*^3/4^. In the velocity dependent cases, the analysis, corroborated by finite-element COMSOL simulations, suggests that entrance flow effects (associated to the curvature of the streamlines at the entrance of the nanochannel), and the vertical drag force exerted by the fluid on the edge of the sheet as it moves upwards, are dominant contributions to the viscous resistance to the motion of the sheet.

For our range of parameters, the critical velocity below which the deformation is quasi-static is about 1 m s^−1^. An estimate for the critical velocity beyond which entrance flow effects make the peeling force velocity dependent was developed (eqn (29)). This estimate is valid provided that the slip length is much larger than the length scale *a*_0_. The dependence of the shape of the sheet on *v* was found to be practically negligible even in the regime where the peeling force displayed a significant dependence on *v*.

For very large peeling velocities, the MD simulations reveal the formation of a cavity near the crack tip. The formation of this cavity was shown to correlate with a decline in the trend of peeling force *vs.* velocity curve. Snapshots from the MD simulations (Fig. 11) show quite evidently that this decline is simply due to the reduction in contact area between the liquid and the solid, so it would be erroneous to associate the reduction in viscous resistance to a variation in slip or viscosity at a critical velocity (cavitation for instance has been used to explain the appearance of sudden slip in atomically smooth mica surface coated with surfactants or self-assembled monolayers^43^).

In the initial stages of peeling of a graphene layer, the linear dimension of the peeled flap can be comparable to the hydrodynamic slip length characterising the liquid-solid interaction (the slip length is a few tens of nanomaters for many graphene solvent combinations^38^). For these small system sizes, the peeling force and the dynamics of the sheets will be controlled by mechanisms similar to the ones discussed in this paper, so our results can be used, for example, to build analytical models to predict thresholds for graphene exfoliation in shear mixing.^14,27,70,75,118^

A possible future development of the current work is the inclusion of thermal fluctuations in the elastic, continuum model. Fluctuations for instance could play an important role in peeling initiation (methods to include the effect of thermal fluctuations in peeling models are available^119–121^). Another relevant avenue of research would be to consider all-atom or coarse-grained MD simulations simulations accounting for longer nanosheets that we were able to simulate here. This could clarify whether a transition from edge- and entrance-dominated viscous dissipation to lubrication-dominated viscous dissipation occurs at a critical value of the crack length.

The current work may have implications for other soft matter systems. Peeling processes have been shown to play an important role in a variety of soft matter problems, from the adhesion of cells^122^ and biological membranes,^123^ to the mechanics of pressure-sensitive adhesives.^124^ Much of the work done on peeling and thin-film lubrication focuses on phenomena occurring near the crack tip,^71^ or in the bulk fluid away from the entrance.^105^ The current work instead highlight the importance of entrance effects, whether associated to the entrance flow or the downward drag on the edge. Entrance effects will be particularly important for soft matter systems such as polymer melts^42^ that can display slip length sometimes exceeding hundreds of microns.^125^ Slip lengths tend to increase as the surface roughness decreases, thus significant slip effects are expected for molecularly smooth surfaces, graphene being a primary example but certainly not the only one.^126^ For both no-slip and slip sheets peeled from a substrate and initially completely bound to it, entrance effects will be more pronounced during the initial stages of peeling, when the interfacial crack is short and the hydrodynamics of the entrance dominates the flow everywhere below the peeled sheet.

## Conflicts of interest

There are no conflicts to declare.

## Supplementary Material

SM-018-D1SM01743H-s001

## References

[cit1] McEwan A. D. (1966). Rheol. Acta.

[cit2] Memet E., Hilitski F., Dogic Z., Mahadevan L. (2021). Soft Matter.

[cit3] Miskin M. Z., Sun C., Cohen I., Dichtel W. R., McEuen P. L. (2018). Nano Lett..

[cit4] Lister J. R., Peng G. G., Neufeld J. A. (2013). Phys. Rev. Lett..

[cit5] Hosoi A. E., Mahadevan L. (2004). Phys. Rev. Lett..

[cit6] Roman B. (2013). Int. J. Fracture.

[cit7] Juel A., Pihler-Puzović D., Heil M. (2018). Annu. Rev. Fluid Mech..

[cit8] Creton C., Ciccotti M. (2016). Rep. Prog. Phys..

[cit9] Andreotti B., Snoeijer J. H. (2020). Annu. Rev. Fluid Mech..

[cit10] Villey R., Creton C., Cortet P.-P., Dalbe M.-J., Jet T., Saintyves B., Santucci S., Vanel L., Yarusso D. J., Ciccotti M. (2015). Soft Matter.

[cit11] Ibarra A., Roman B., Melo F. (2016). Soft Matter.

[cit12] Dillard D. A., Mukherjee B., Karnal P., Batra R. C., Frechette J. (2018). Soft Matter.

[cit13] Cao Q., Geng X., Wang H., Wang P., Liu A., Lan Y., Peng Q. (2018). Crystals.

[cit14] Stafford J., Uzo N., Farooq U., Favero S., Wang S., Chen H.-H., L’Hermitte A., Petit C., Matar O. K. (2021). 2D Mater..

[cit15] Chen X., Boulos R. A., Dobson J. F., Raston C. L. (2013). Nanoscale.

[cit16] Schneider G. F., Calado V. E., Zandbergen H., Vandersypen L. M., Dekker C. (2010). Nano Lett..

[cit17] Seo J., Kim C., Ma B. S., Lee T.-I., Bong J. H., Oh J. G., Cho B. J., Kim T.-S. (2018). Adv. Funct. Mater..

[cit18] Li B., Klekachev A. V., Cantoro M., Huyghebaert C., Stesmans A., Asselberghs I., De Gendt S., De Feyter S. (2013). Nanoscale.

[cit19] Wu J., Xie L., Li Y., Wang H., Ouyang Y., Guo J., Dai H. (2011). J. Am. Chem. Soc..

[cit20] Annett J., Cross G. L. (2016). Nature.

[cit21] Ishikawa M., Ichikawa M., Okamoto H., Itamura N., Sasaki N., Miura K. (2012). Appl. Phys. Express.

[cit22] Sinclair R. C., Suter J. L., Coveney P. V. (2018). Adv. Mater..

[cit23] Ke C., Zheng M., Zhou G., Cui W., Pugno N., Miles R. N. (2010). Small.

[cit24] Bordes É., Szala-Bilnik J., Pádua A. A. (2018). Faraday Discuss..

[cit25] Shih C. J., Lin S., Strano M. S., Blankschtein D. (2010). J. Am. Chem. Soc..

[cit26] Sresht V., Pádua A. A., Blankschtein D. (2015). ACS Nano.

[cit27] Salussolia G., Barbieri E., Pugno N. M., Botto L. (2020). J. Mech. Phys. Solids.

[cit28] Ball T. V., Neufeld J. A. (2018). Phys. Rev. Fluids.

[cit29] Kannam S. K., Todd B., Hansen J. S., Daivis P. J. (2011). J. Chem. Phys..

[cit30] Calabrò F., Lee K., Mattia D. (2013). Appl. Mathematics Lett..

[cit31] Kotsalis E. M., Walther J. H., Koumoutsakos P. (2004). Int. J. Multiphase Flow.

[cit32] Neek-Amal M., Lohrasebi A., Mousaei M., Shayeganfar F., Radha B., Peeters F. (2018). Appl. Phys. Lett..

[cit33] Wang F.-C., Zhao Y.-P. (2011). Soft Matter.

[cit34] Neek-Amal M., Peeters F. M., Grigorieva I. V., Geim A. K. (2016). ACS Nano.

[cit35] Horn H. W., Swope W. C., Pitera J. W., Madura J. D., Dick T. J., Hura G. L., Head-Gordon T. (2004). J. Chem. Phys..

[cit36] Zhang H., Ye H., Zheng Y., Zhang Z. (2011). Microfluid. Nanofluid..

[cit37] Werder T., Walther J. H., Jaffe R., Halicioglu T., Koumoutsakos P. (2003). J. Phys. Chem. B.

[cit38] Kamal C., Gravelle S., Botto L. (2020). Nat. Commun..

[cit39] Maali A., Cohen-Bouhacina T., Kellay H. (2008). Appl. Phys. Lett..

[cit40] Ortiz-Young D., Chiu H.-C., Kim S., Votchovsky K., Riedo E. (2013). Nat. Commun..

[cit41] Tocci G., Joly L., Michaelides A. (2014). Nano Lett..

[cit42] Hatzikiriakos S. G. (2015). Soft Matter.

[cit43] Zhu Y., Granick S. (2001). Phys. Rev. Lett..

[cit44] Zhu Y., Granick S. (2002). Macromolecules.

[cit45] Hemadri V., Varade V. V., Agrawal A., Bhandarkar U. (2017). Phys. Fluids.

[cit46] Falk K., Sedlmeier F., Joly L., Netz R. R., Bocquet L. (2010). Nano Lett..

[cit47] Lhermerout R., Perkin S. (2018). Phys. Rev. Fluids.

[cit48] Plimpton S. (1995). J. Comput. Phys..

[cit49] Abascal J. L., Vega C. (2005). J. Chem. Phys..

[cit50] Stuart S. J., Tutein A. B., Harrison J. A. (2000). J. Chem. Phys..

[cit51] Gravelle S., Joly L., Ybert C., Bocquet L. (2014). J. Chem. Phys..

[cit52] Marchio S., Meloni S., Giacomello A., Valeriani C., Casciola C. (2018). J. Chem. Phys..

[cit53] Ouyang W., Hod O., Urbakh M. (2021). ACS Appl. Mater. Interfaces.

[cit54] González M. A., Abascal J. L. (2010). J. Chem. Phys..

[cit55] Holz M., Heil S. R., Sacco A. (2000). Phys. Chem. Chem. Phys..

[cit56] Landau L. D. and Lifšic E. M., Theory of elasticity: volume 7, Elsevier, 1986, vol. 7

[cit57] Jones J. E. (1924). Proc. R. Soc. London, Ser. A.

[cit58] Kelly B. T. , Physics of graphite, Applied Science, 1981, p. 477

[cit59] Okamoto R., Yamasaki K., Sasaki N. (2018). Mater. Chem. Front..

[cit60] Ghatak A., Mahadevan L., Chaudhury M. K. (2005). Langmuir.

[cit61] Mosaddeghi H., Alavi S., Kowsari M., Najafi B. (2012). J. Chem. Phys..

[cit62] Gravelle S., Ybert C., Bocquet L., Joly L. (2016). Phys. Rev. E.

[cit63] Pihler-Puzović D., Juel A., Peng G. G., Lister J. R., Heil M. (2015). J. Fluid Mech..

[cit64] Wang Z.-Q., Detournay E. (2018). J. Appl. Mech..

[cit65] Lister J. R. (1990). J. Fluid Mech..

[cit66] Kierzenka J., Shampine L. F. (2001). ACM Transactions on Mathematical Software (TOMS).

[cit67] Kendall K. (1975). J. Adhesion.

[cit68] Young Y. N., Stone H. A. (2017). Phys. Rev. Fluids.

[cit69] Obreimoff J. (1930). Proc. R. Soc. London, Ser. A.

[cit70] Botto L. (2019). Front. Mater..

[cit71] Rieutord F., Bataillou B., Moriceau H. (2005). Phys. Rev. Lett..

[cit72] Ghatak A., Chaudhury M. K. (2003). Langmuir.

[cit73] Ghatak A., Mahadevan L., Chung J. Y., Chaudhury M. K., Shenoy V. (2004). Proc. R. Soc. London, Ser. A.

[cit74] Israelachvili J. N. , Intermolecular and surface forces, Academic press, 2015

[cit75] Gravelle S., Kamal C., Botto L. (2020). J. Chem. Phys..

[cit76] Wang J., Sorescu D. C., Jeon S., Belianinov A., Kalinin S. V., Baddorf A. P., Maksymovych P. (2016). Nat. Commun..

[cit77] van Engers C. D., Cousens N. E., Babenko V., Britton J., Zappone B., Grobert N., Perkin S. (2017). Nano Lett..

[cit78] Novoselov K., Mishchenko O. A., Carvalho O. A., Neto A. C. (2016). Science.

[cit79] Nocedal J. and Wright S. J., Nonlinear Equations, Numerical Optimization, Springer New York, 2006, pp. 270–302

[cit80] Wang Q. (2010). Phys. Lett. A.

[cit81] Kang J. W., Lee S. (2013). Comput. Mater. Sci..

[cit82] Dillard D. (1989). J. Appl. Mech..

[cit83] Glassmaker N., Hui C. (2004). J. Appl. Phys..

[cit84] Wagner T. J., Vella D. (2013). Soft Matter.

[cit85] Lister J. R., Skinner D. J., Large T. M. (2019). J. Fluid Mech..

[cit86] Dhong C., Fréchette J. (2017). J. Appl. Phys..

[cit87] Pihler-Puzović D., Peng G. G., Lister J. R., Heil M., Juel A. (2018). J. Fluid Mech..

[cit88] Cross B., Barraud C., Picard C., Léger L., Restagno F., Charlaix É. (2018). Phys. Rev. Fluids.

[cit89] Münch A. (2005). J. Phys.: Condens. Matter.

[cit90] Münch A., Wagner B., Witelski T. P. (2005). J. Eng. Math..

[cit91] Brochard-Wyart F., De Gennes P.-G., Hervert H., Redon C. (1994). Langmuir.

[cit92] Desroches J., Detournay E., Lenoach B., Papanastasiou P., Pearson J. R.-A., Thiercelin M., Cheng A. (1994). Proc. R. Soc. London, Ser. A.

[cit93] Chan T. S., Kamal C., Snoeijer J. H., Sprittles J. E., Eggers J. (2020). J. Fluid Mech..

[cit94] Huh C., Scriven L. (1971). J. Colloid Interface Sci..

[cit95] Sui Y., Ding H., Spelt P. D. (2014). Annu. Rev. Fluid Mech..

[cit96] Barrat J.-L., Bocquet L. (1999). Phys. Rev. Lett..

[cit97] Bakli C., Chakraborty S. (2019). Nanoscale.

[cit98] Bocquet L., Charlaix E. (2010). Chem. Soc. Rev..

[cit99] Lauga E. , Brenner M. and Stone H., Microfluidics: The no-slip boundary condition, Springer Handbook of Experimental Fluid Mechanics, Springer Berlin Heidelberg, 2007, pp. 1219–1240

[cit100] Hasimoto H. (1958). J. Phys. Soc. Jpn..

[cit101] Gravelle S., Joly L., Detcheverry F., Ybert C., Cottin-Bizonne C., Bocquet L. (2013). Proc. Natl. Acad. Sci. U. S. A..

[cit102] Belin C., Joly L., Detcheverry F. (2016). Phys. Rev. Fluids.

[cit103] Happel J. and Brenner H., Low Reynolds number hydrodynamics: with special applications to particulate media, Springer Science & Business Media, 2012, vol. 1

[cit104] Jensen K. H., Valente A. X., Stone H. A. (2014). Phys. Fluids.

[cit105] Bäumchen O., Marquant L., Blossey R., Münch A., Wagner B., Jacobs K. (2014). Phys. Rev. Lett..

[cit106] Eggers J. (1997). Rev. Mod. Phys..

[cit107] Thoroddsen S. T., Nguyen H., Takehara K., Etoh T. (2010). Phys. Rev. E: Stat., Nonlinear, Soft Matter Phys..

[cit108] Biddle J. W., Singh R. S., Sparano E. M., Ricci F., González M. A., Valeriani C., Abascal J. L., Debenedetti P. G., Anisimov M. A., Caupin F. (2017). J. Chem. Phys..

[cit109] González M. A., Valeriani C., Caupin F., Abascal J. L. (2016). J. Chem. Phys..

[cit110] Bengtsson S., Ljungberg K., Vedde J. (1996). Appl. Phys. Lett..

[cit111] Peng G. G., Lister J. R. (2020). J. Fluid Mech..

[cit112] Roman B., Bico J. (2010). J. Phys.: Condens. Matter.

[cit113] Frisch-Fay R. , Flexible bars, Butterworths, 1962, p. 220

[cit114] Audoly B. , Introduction to the Elasticity of Rods, Fluid-Structure Interactions in Low-Reynolds-Number Flows, The Royal Society of Chemistry, 2015, pp. 1–24

[cit115] Lindner A. and Shelley M., Elastic Fibers in Flows, Fluid-Structure Interactions in Low-Reynolds-Number Flows, The Royal Society of Chemistry, 2016, pp. 168–192

[cit116] Jabbarzadeh M., Fu H. C. (2020). J. Comput. Phys..

[cit117] Shi X., Peng B., Pugno N. M., Gao H. (2012). Appl. Phys. Lett..

[cit118] Li Z., Young R. J., Backes C., Zhao W., Zhang X., Zhukov A. A., Tillotson E., Conlan A. P., Ding F., Haigh S. J. (2020). et al.. ACS Nano.

[cit119] Qian J., Lin J., Xu G.-K., Lin Y., Gao H. (2017). J. Mech. Phys. Solids.

[cit120] Zhao C., Sprittles J. E., Lockerby D. A. (2019). J. Fluid Mech..

[cit121] Zhang Y., Sprittles J. E., Lockerby D. A. (2019). Phys. Rev. E.

[cit122] Décavé E., Garrivier D., Bréchet Y., Bruckert F., Fourcade B. (2002). Phys. Rev. Lett..

[cit123] de Gennes P-G. , Adhesion of soft objects, The Physics of Complex Systems (New Advances and Perspectives), IOS Press, 2004, pp. 3–15

[cit124] Perrin H., Eddi A., Karpitschka S., Snoeijer J. H., Andreotti B. (2019). Soft Matter.

[cit125] Léger L., Hervet H., Massey G., Durliat E. (1997). J. Phys.: Condens. Matter.

[cit126] Granick S., Zhu Y., Lee H. (2003). Nat. Mater..

[cit127] King T. , Butcher S. and Zalewski L., Apocrita - High Performance Computing Cluster for Queen Mary University of London, 2017

