# Supplementary material for: Viscous peeling of a nanosheet
Source: Soft Matter. 2022 May 5;18(20):3967–80. doi: 10.1039/d1sm01743h (PMC9131316; doi:10.1039/d1sm01743h)
Supplement: SM-018-D1SM01743H-s001 [file SM-018-D1SM01743H-s001.pdf]

Electronic Supplementary Material (ESI) for Soft Matter.
This journal is © The Royal Society of Chemistry 2022

Supplementary Information for
"Viscous peeling of a nanosheet"

Adyant Agrawal[(a)], Simon Gravelle[(a)], Catherine Kamal[(a)], Lorenzo Botto[(b)]

[a] *School of Engineering and Material Science, Queen Mary University of London, London, United Kingdom*
[b] *Process and Energy Department, 3ME Faculty of Mechanical, Maritime and Materials Engineering, TU Delft, Delft, The Netherlands.*

1 Peeling force vs edge height from the continuum model

We use eq.(27) in the main article to calculate the pressure drop at entrance and COMSOL simulations to calculate the edge drag force (see Fig.10a in the main article). The edge drag force is applied as a concentrated force at the left edge.

The increase in F with v is qualitatively similar to that suggested by the MD data. The agreement is not perfect because these continuum estimated of entrance pressure drop and drag force are 1D approximations for small deflections of the sheet.

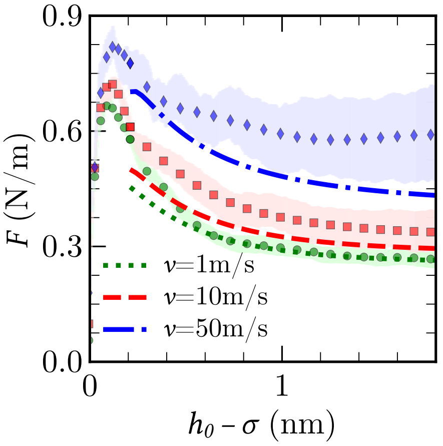

Figure S1: Peeling force as a function of edge height computed from the continuum model for different peeling velocities (continuous lines). The symbols represent MD data; $v = 1\,\text{m/s}$ (green disks), $v = 10\,\text{m/s}$ (red squares), and $v = 50\,\text{m/s}$ (blue diamonds).

2 Molecular dynamics details

2.1 Method

We use the Adaptive Intermolecular Reactive Empirical Bond Order (AIREBO) force field to model graphene[1], the TIP4P/2005 model for water[2] and the all-atom Gromos force field for NMP[3]. Carbon-water interaction parameters are calculated using the Lorentz-Berthelot mixing rule. Water molecules are maintained at a constant temperature $T = 300\,\text{K}$ using a Nosé-Hoover temperature thermostat[4,5].

2.2 Peeling in vacuum

We have carried out simulations of peeling of graphene in vacuum for $v = 0$ (quasi-steady case) and $v = 100\,\text{m/s}$. Fig. S2 shows that there is no difference in F for the two velocities in the case of vacuum. Contrarily, the increase in force is evident for water at $v = 100\,\text{m/s}$.

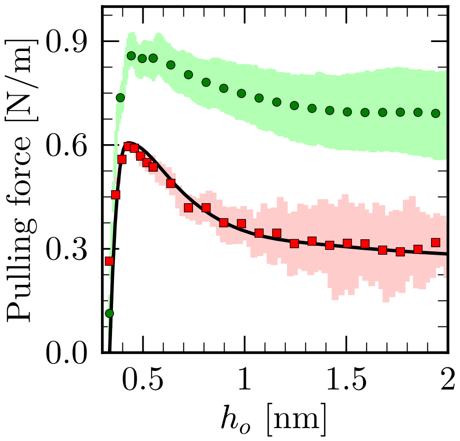

Figure S2: Peeling force as a function of edge height in MD for $v = 100\,\mathrm{m/s}$ in vacuum (red squares); $v = 0$ in vacuum (solid line); $v = 100\,\mathrm{m/s}$ in water (green discs). The colored areas correspond to standard deviation.

2.3 Peeling in NMP solvent

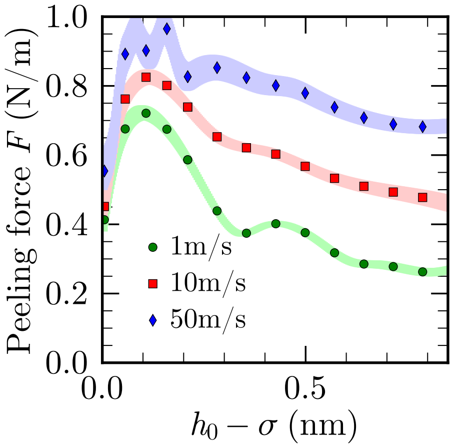

Figure S3: Peeling force as a function of edge height in MD for different peeling velocities. The colored areas correspond to standard deviation.

We have performed peeling simulations of graphene in N-Methyl-2-pyrrolidone (NMP) for v =1, 10 and 50 m/s. We find that the F vs h_0 shows characteristic similarities to the case of water (Fig.S3). Interestingly, we notice a wavy pattern in the case of NMP. Shih *et al.* have recently reported that the potential between graphene sheets exhibit an oscillatory pattern in NMP [6]. The large size of NMP molecules can also cause nanofluidic effects, for example, fluid structuring or disjoining pressure [7].

3 A COMSOL analysis of the entrance pressure drop for 2D circular entrances with infinite-slip walls

We have carried out COMSOL simulations for a system in which fluid from a reservoir is flowing into a stationary channel of width a through a circular entrance with a constant radius of curvature R, as shown in the inset of Fig. S4. In these simulations, we solve the incompressible Stokes equations with free slip boundary condition at all surfaces. We impose a flux Q at the right end of the channel. The reservoir height and width of the computational domain is $100R \times 100R$ to avoid finite size effect. We measure the pressure difference (ΔP) between the far edge of the reservoir and the channel outlet. We plot $\Delta P/P_0$ as a function of R/a (Fig. S4) where P_0 is the pressure difference given by Hasimoto's formula [8] (Eq.24).

We analytically solve for the pressure drop in the converging channel. Using Stokes equation at the

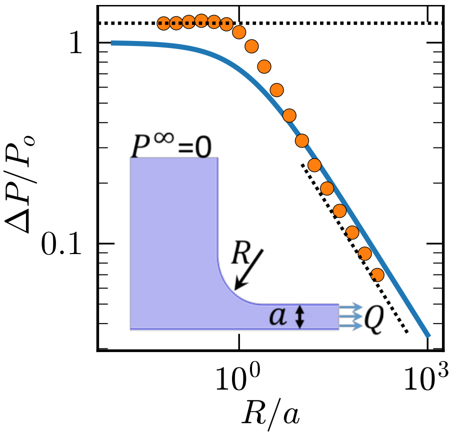

Figure S4: $\Delta P/P_o$ as a function of R/a from COMSOL (orange discs). The solid, blue line represents solution of eq.S3. Slanted dotted line represents $y = x^{-1/2}$ and the horizontal dotted line represents $y = 1.25$. Inset shows schematic of the system.

converging channel wall, $\nabla^2 u_x \neq 0$ and hence $p_x \neq 0$, where u_x is the local fluid velocity in x-direction. Using dimensional arguments, the velocity gradient can be estimated as $|\nabla^2 u_x| \sim U(x)/\delta^2(x)$, where $U(x) = Q/(h-d)$ is the y-averaged fluid velocity below the sheet and $\delta(x)$ is the local length scale over which $U(x)$ varies. Thus, a simple model for the horizontal pressure gradient is

$$\frac{dp}{dx} \simeq \frac{\mu Q}{(h-d)\delta^2(x)}. \tag{S1}$$

It is expected that this pressure gradient is a function of the curvature of the channel[9]. The pressure drop corresponding to eq.S1,

$$\Delta P \simeq \mu Q \int_0^R \frac{1}{h\delta^2(x)} dx \tag{S2}$$

We know from Hasimoto's result that $\lim_{R\to 0} \Delta P \simeq -8\mu Q/\pi a^2$. This suggests that $\delta^2(x) \simeq -\pi h R/8$ as $h \to a$ for $R \to 0$, thus

$$\Delta P \simeq -\frac{8\mu Q}{\pi} \int_0^R \frac{1}{h^2 R} dx. \tag{S3}$$

We compare the results of Eq.S3 with the COMSOL simulations described above in Fig. S4. Both the analytical and numerical results show a plateau for $R/a \leq 1$, approximately, and a region for $R/a > 1$ for which ΔP decays. The decay rate predicted by COMSOL results for large values of R/a is very close to the one found numerically using Eq.S3, i.e., for $R/a \gg 1$, $\Delta P/P_0$ follows the power-law $(R/a)^{-1/2}$. For $R/a \leq 1$, the COMSOL results for $\Delta P/P_0$ are a 25 % larger than the numerical prediction as expected[9,10], i.e., for a costant Q, ΔP predicted by Hasimoto's formula (Eq.24) is nearly 20% lower in magnitude than ΔP predicted using COMSOL.

References

[1] S. J. Stuart, A. B. Tutein and J. A. Harrison, *The Journal of chemical physics*, 2000, **112**, 6472–6486.

[2] J. L. Abascal and C. Vega, *The Journal of chemical physics*, 2005, **123**, 234505.

[3] N. Schmid, A. P. Eichenberger, A. Choutko, S. Riniker, M. Winger, A. E. Mark and W. F. van Gunsteren, *European biophysics journal*, 2011, **40**, 843–856.

[4] S. Nosé, *Molecular physics*, 1984, **52**, 255–268.

[5] W. G. Hoover, *Physical review A*, 1985, **31**, 1695.

[6] C. J. Shih, S. Lin, M. S. Strano and D. Blankschtein, *Journal of the American Chemical Society*, 2010, **132**, 14638–14648.

[7] M. Neek-Amal, A. Lohrasebi, M. Mousaei, F. Shayeganfar, B. Radha and F. Peeters, *Applied Physics Letters*, 2018, **113**, 083101.

[8] H. Hasimoto, *Journal of the Physical Society of Japan*, 1958, **13**, 633–639.

[9] C. Belin, L. Joly and F. Detcheverry, *Physical Review Fluids*, 2016, **1**, 054103.

[10] S. Gravelle, L. Joly, F. Detcheverry, C. Ybert, C. Cottin-Bizonne and L. Bocquet, *Proceedings of the National Academy of Sciences*, 2013, **110**, 16367–16372.
